# Drivers and functional consequences of covering in a pervasive marine grazer (green sea urchin, *Strongylocentrotus droebachiensis*)

**DOI:** 10.1186/s12983-026-00616-3

**Published:** 2026-05-29

**Authors:** Chantelle R. Clermont, Patrick Gagnon

**Affiliations:** Department of Ocean Sciences, Ocean Sciences Centre, Memorial University of Newfoundland, St. John’s, Newfoundland A1C 5S7 Canada

**Keywords:** Behaviour, Echinoderm, Green sea urchin, Covering material, Displacement, Wave action, Hydrodynamic forces, Body size, Light, Mechanical protection

## Abstract

**Background:**

The circumpolar green sea urchin, *Strongylocentrotus droebachiensis,* exhibits a fascinating behaviour, termed “covering”, which consists of coating the body with materials collected in the environment. The lack of a consensus about the drivers of covering in green sea urchin and their relative importance prevents accurate predictions about the frequency and intensity of the behaviour and its functional consequences in a globally changing ocean climate. We paired an experiment in an oscillatory wave tank with green sea urchins collected from an extensive sea urchin barren in southeastern Newfoundland (Canada), and a 3-mo survey of this barrens, to examine individual and interactive effects of wave action, light, sea urchin body size, and types of covering materials on sea urchin’s displacement and covering.

**Results:**

Our findings establish that covering in *S. droebachiensis* is: (a) predominantly controlled by hydrodynamic forces, with the existence of water current-induced covering tipping points; (b) ontogenetically determined, with a continuous inclination to cover in small individuals and a seasonal component to covering in larger individuals; (c) opportunistic, with multiple types of covering materials employed based on availability; and (d) functionally costly, as it significantly reduces mobility. We largely rule out the paradigm that light induces covering or at least clearly marginalize it as a trigger or effector. Our results and those of other studies of the behavioural repertoire of *S. droebachiensis*, are consistent with the notion that covering serves a complementary function to mitigation of hydrodynamic forces.

**Conclusions:**

We propose that covering in *S. droebachiensis* primarily serves a mechanical protection function, whereby the species shields its body surface to protect its external sensory, defensive, and locomotory organs against physical contact with moving debris.

**Supplementary information:**

The online version contains supplementary material available at 10.1186/s12983-026-00616-3.

## Introduction

Marine organisms exhibit a variety of behaviours that are often influenced by environmental conditions and vary with life-history stages [1, 2]. In eastern Canada, green sea urchin, *Strongylocentrotus droebachiensis*, dominates shallow rocky subtidal habitats, where it aggregates into grazing fronts that create areas with low seaweed abundance, termed urchin barrens, as individuals advance through and consume kelp beds [3–5]. Larger green sea urchins typically dominate grazing fronts, whereas smaller individuals primarily inhabit kelp beds [5–7], though this pattern may vary with seasonal shifts in wave-induced sea urchin microhabitat selection, sea temperature, and grazing ability [6, 8, 9]. These shifts are more frequent in larger individuals and vary with seasonal temperature changes [6, 10], yet they appear to be predominantly driven by wave velocity in habitats with moderate to high hydrodynamic forces, as seen in other benthic marine organisms [9, 11–13]. When exposed to wave action, the species can, depending on population density, also reduce displacement and feeding, while selecting microhabitats that facilitate clinging [8, 9]. These behaviours have presumably evolved to mainly minimize the risk of dislodgement, physical damage (e.g. abrasion), and algal whiplash caused by wave action [8, 14–16]. Even though light attenuates quickly with increasing depth in shallow subtidal environments, it can lead to cellular damage and stress in sea urchins while also impacting their distribution and behaviour [17, 18]. With a changing ocean climate, coastal regions are predicted to experience more frequent and intense wave action, as well as increased light exposure, potentially altering behaviour in many ecologically important species [19–21].

Sea urchins, including *S. droebachiensis*, exhibit a fascinating behaviour termed “covering”, which consists in coating the body with materials collected in the environment [22–25]. This behaviour also exists in species from other phyla, including decorator crabs in the Majoidea superfamily, some insect larvae, and large ungulates [22–24]. Sea urchins typically cover by picking up and holding organic and inorganic materials (debris) on their aboral surface with their tube feet [25–27]. Possible reasons for covering in sea urchins are unclear and may include reduction of the risks of predation or dislodgement by wave action or some biological aversion to sunlight, in particular ultraviolet radiation (UVR) [24, 28, 29]. The factors that drive the frequency and intensity of this behaviour also remain largely unexplored and ill-defined, in part because they may also vary temporally and intra- and inter-specifically [30–32].

Despite the lack of direct measurements, covering presumably involves energetic costs and hence may limit the ability to perform critical functions such as aggregation, foraging, and displacement [7, 30, 32]. Several studies suggest that covering in *S. droebachiensis* increases with exposure to UVA and UVB while varying with body size, and that UVR may have less of an impact than wave surge and algal whiplash [24, 28, 33]. Dumont et al. (2007) [24] found no significant relationship between covering and the presence of predators. Covering materials used by green sea urchin in eastern Canada typically include macroalgae, mussel shells, and pebbles [24], which the species may actively select [24, 34, 35], as may also be the case in other sea urchin species [31, 36]. The chronic lack of knowledge and consensus about the drivers of covering in green sea urchin and their relative importance prevents accurate predictions about the frequency and intensity of the behaviour and how that may change in a globally shifting ocean climate [19, 20]. Well-replicated experiments under controlled environmental conditions, and surveys in natural habitats are required to establish accurate relationships between green sea urchin covering and environmental variability and their implications for the ecology of the species.

In the present study we paired an experiment in an oscillatory wave tank with green sea urchins collected from an extensive sea urchin barren in southeastern Newfoundland (Canada), and a 3-mo survey of the latter barrens, to examine individual and interactive effects of sea urchin body size, light conditions, wave action, and type of covering material on sea urchin’s displacement and covering. In the wave tank experiment, we exposed sea urchins of two body sizes (small, large) to orthogonal combinations of three light conditions (dark, white light, UVR + white light) and three wave velocities (null, low, intermediate). We characterized the distance the urchins moved and the frequency and intensity of covering with two types of materials common in barrens (mussel shell pieces, rhodolith fragments). We predicted that (1) displacement would be inversely related to covering, whereas the frequency and intensity of covering would be (2) higher in the presence of UVR, (3) directly related to wave velocity, and (4) inversely related to body size. In the barrens, we tracked changes from late May to early September in the frequency and intensity of covering with four prominent types of materials (mussel shell pieces, rhodolith fragments, kelp, and “other”) at each of three depths (3, 5, 9 m). We predicted that the frequency of covering would be (5) inversely related to depth, while (6) varying seasonally, with a generally (7) stronger response in small than large individuals. These predictions stem from the literature cited above about covering in *S. droebachiensis* or other sea urchin species [24, 28, 33], and from other studies of the impacts of environmental variability (e.g. wave action, sea temperature, light) on displacement, aggregation, and feeding in Newfoundland *S. droebachiensis* [6, 8, 37, 38].

## Materials and methods

### Study and collection site

**Fig. 1:**
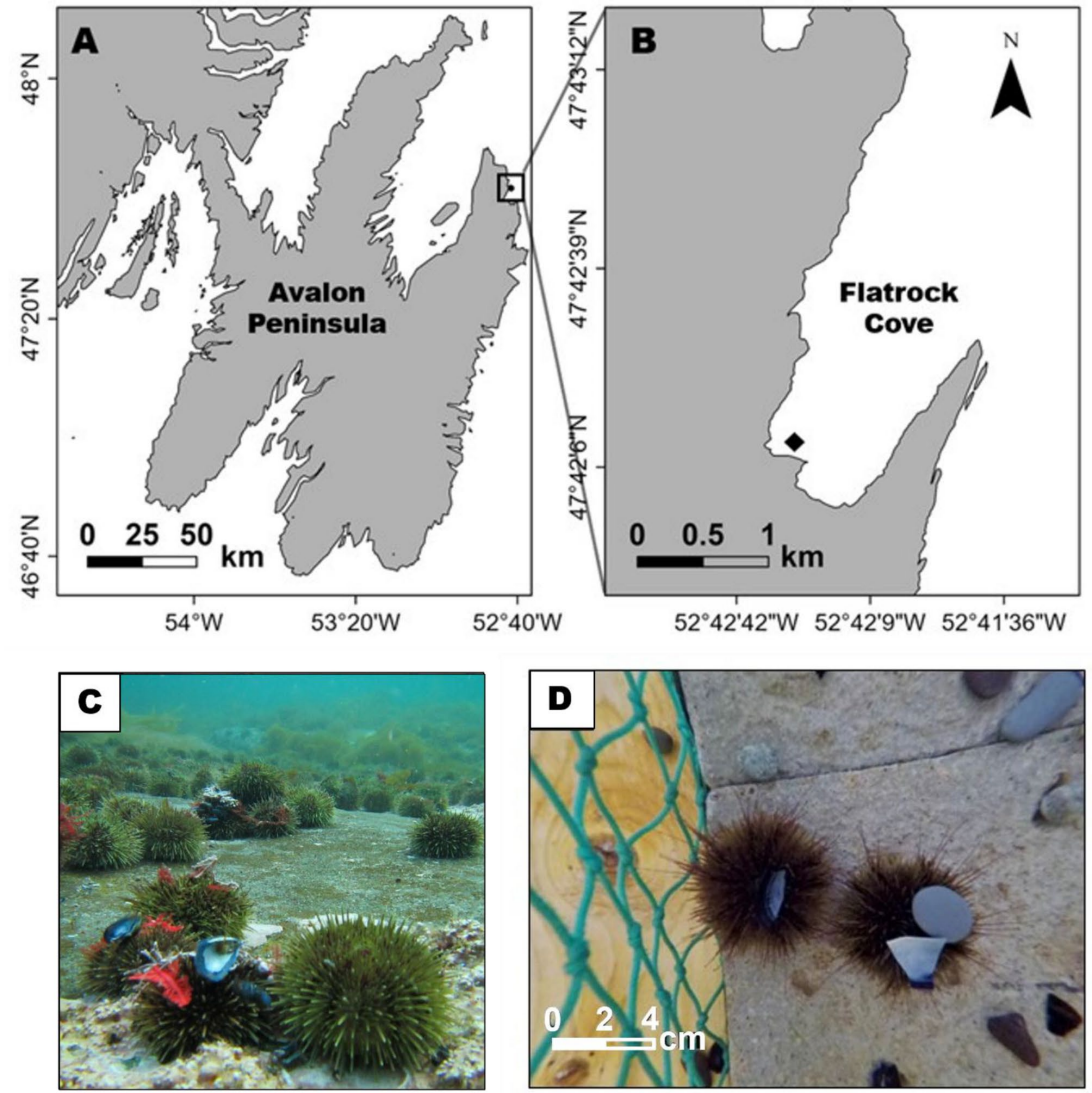
(**A**) Avalon Peninsula [southeastern Newfoundland, Canada] and (**B**) location of the study and collection site [solid diamond] in Flatrock Cove [FC]. Green sea urchins (*Strongylocentrotus droebachiensis*) exhibiting covering in the (**C**) sea urchin barrens in FC (photo credit: S. Trueman), and (**D**) wave tank used in the present study (see Sect. “Wave tank experiment”)

The present study was carried out with green sea urchins (*Strongylocentrotus droebachiensis*) located at, or collected from, Flatrock Cove (FC) in southeastern Newfoundland, Canada (47° 35’ 30.9” N, 52° 53’ 35.2” W) (Fig. 1). FC is a relatively large (~2 km^2^) embayment exposed to northeasterly winds and waves. The seabed at the study and collection site is composed of gently sloping bedrock, to a depth of ~15 m, with scattered boulders below ~10 m. Kelp beds, mainly *Alaria esculenta* and *Laminaria digitata*, dominate the 0–3 m depth range during spring and summer, followed in deeper water by an extensive green sea urchin barren.

### Collection and acclimation

Scuba divers hand-collected 360 small (1 to 2 cm in test diameter; t.d.) and 360 large (4 to 5 cm t.d.) green sea urchins at the FC site at depths between 5 and 9 m on 3 July, 2016. These sizes were chosen to reflect the smallest and largest size classes most frequently observed at this location. Sea urchins were transported in large containers filled with seawater from FC to the Ocean Sciences Centre (OSC) of Memorial University of Newfoundland (MUN). Upon arrival at the OSC (<5 h after collection), we transferred the sea urchins to 75-L holding tanks supplied with ambient flow-through seawater pumped in from a depth of ~5 m from the adjacent embayment, Logy Bay. Each tank contained 75 to 100 large, or 300 small, individuals held there for five days without food to standardize hunger levels. Feces were siphoned out of the tanks twice during this acclimation period.

After the 5-d acclimation, we fed sea urchins with fresh *A. esculenta* cut into 2.5 × 2.5-cm pieces (~12.5 g wet weight per holding tank) and cleaned the tanks once every two days. Water temperature in the tanks varied minimally, averaging 6.9°C. Sea urchins were exposed to ambient light that came in through windows in the lab. Sea urchins were used in the experiment (see Sect. “Wave tank experiment”) within 2–3 weeks of collection.

### Wave tank experiment

**Fig. 2:**
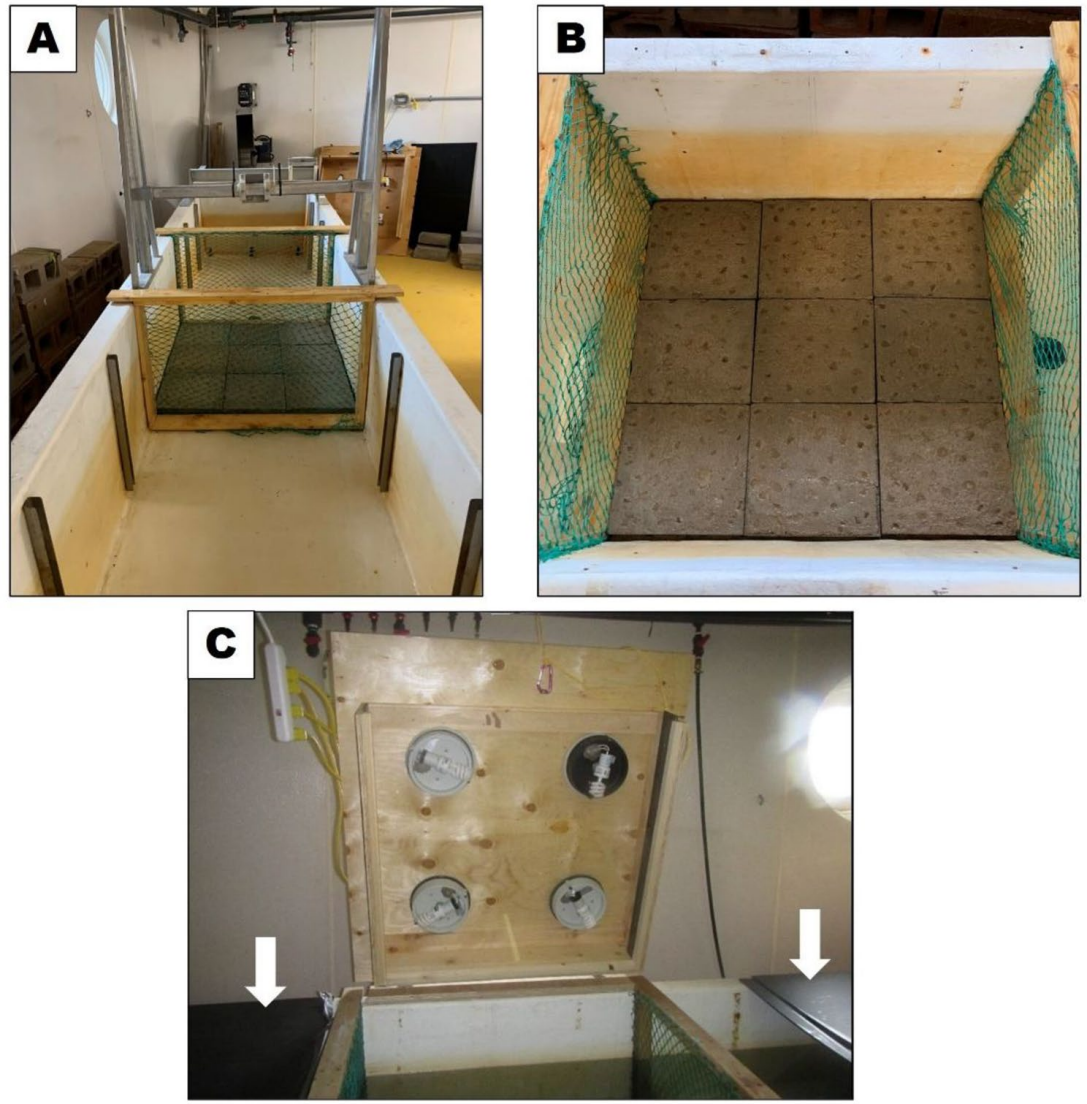
(**A**) Oscillatory wave tank with experimental area [0.9 (L) x 0.9 (W) x 0.6 (H) m] delimited in its centre with 2.5-cm mesh nylon netting. The large aluminum frame sitting on the sides of the tank held the digital camera used to take images of the experimental area at set intervals during trials. (**B**) Close-up view of the experimental area [viewed from above] with nine corrugated concrete tiles [30 (L) x 30 (W) x 0.5 (H) cm each] that mimicked natural seabed heterogeneity. (**C**) Cover with integrated lighting system (four light bulbs; Eco Terra Repti 6 glow, dessert) used to create the six UVR-based experimental treatments (Table 1) in the wave tank experiment. The cover, positioned right above the experimental area, is upright for viewing purposes but otherwise facing down during the trials. Blackout sheets (identified with arrows) were placed above the rest of the wave tank to block external light input

To investigate the impacts of wave action and UV Radiation (UVR) on covering, we ran an experiment in an oscillatory wave tank that produces a bidirectional flow of seawater similar to that experienced by organisms in shallow subtidal habitats. The wave tank system is a more advanced and automated version of that initially developed by Gagnon et al. [39], and more fully described in [6, 8, 40]. It essentially consists in a 6-m long through in which seawater is pushed back and forth at an adjustable, yet constant speed, by a panel hinged to the bottom of the tank at one end of the through. The top of the panel is attached to a metal rod welded to the shaft of an electric motor whose rotational speed is controlled finely by a computer (Fig. 2A). This particular design, whereby the top of the rotating panel pushes the water back and forth, allows for the build-up and maintenance of a progressive, sinusoidal wave in the centre of the tank, i.e. where the experimental area is located, as demonstrated with time series measurements (see Fig. 4 in [39]) of flow velocity 5 cm above the bottom of the experimental area with a Doppler current meter (Vector Current Meter; Nortek). We ran the experiment over ~2 weeks in July of 2016 when seawater in the wave tank was sufficiently warm to promote sustained and consistent activity levels in *S. droebachiensis* [8]. After the 5-d acclimation period, we exposed sea urchins to various wave velocities and light conditions to determine effects on covering. Experimental treatments, 18 in total, were orthogonal combinations of two sea urchin body size classes: small (S; 1 to 2 cm t.d.) and large (L; 4 to 5 cm t.d.), three light conditions: dark (D, 0.02 μmol m^−2^ s^−1^; used as a control), white light (W, 5.83 μmol m^− 2^ s^− 1^; representing ambient light), and UVR + white light (U, 6.29 μmol m^−2^ s^−1^; representing solar radiation), and three wave velocities: null (N, 0 m s^−1^; still water), low (L, 0.1 m s^−1^), and intermediate (I, 0.2 m s^−1^) (Table 1, see description of UV-emitting light bulbs in the paragraph below).

**Table 1:** Sea urchin body size (small [S]: 1 to 2 cm, large [L]: 4 to 5 cm t.D.), light condition (dark [D]: 0.02, white light [W]: 5.83, UVR + white light [U]: 6.29 μmol m^−2^ s^−1^), and wave velocity (null [N]: 0, low [L]: 0.1, intermediate [I]: 0.2 m s^−1^) for each treatment tested in the oscillatory wave tank experiment

Treatment ID	Body size	Light condition	Wave velocity
SDN	Small	Dark	Null
SDL	Small	Dark	Low
SDI	Small	Dark	Intermediate
SWN	Small	White light	Null
SWL	Small	White light	Low
SWI	Small	White light	Intermediate
SUN	Small	UVR + white light	Null
SUL	Small	UVR + white light	Low
SUI	Small	UVR + white light	Intermediate
LDN	Large	Dark	Null
LDL	Large	Dark	Low
LDI	Large	Dark	Intermediate
LWN	Large	White light	Null
LWL	Large	White light	Low
LWI	Large	White light	Intermediate
LUN	Large	UVR + white light	Null
LUL	Large	UVR + white light	Low
LUI	Large	UVR + white light	Intermediate

We enclosed the centre of the wave tank (0.9 m length × 0.9 m width × 0.6 m height) with 2.5-cm mesh nylon netting for use as the experimental area (Fig. 2A). We used this netting to keep sea urchins within the experimental area and to ensure they could not fit through the lattices while enabling unobstructed water flow [41]. Nine 0.3 × 0.3-m corrugated concrete tiles were placed on the bottom of the experimental area to mimic the heterogeneity of the natural seabed in shallow subtidal habitats (Fig. 2B). The same quantities of mussel shell pieces and rhodolith fragments were used simultaneously as covering material in each trial. Mussel shell pieces consisted of 290 g of blue mussel (*Mytilus edulis*) shell pieces of 1 to 2 cm^2^. Rhodolith fragments consisted of 550 g of rhodolith (*Boreolithothamnion glaciale*) fragments of 1 to 2 cm^2^. We chose these materials because *S. droebachiensis* uses both commonly as covering material in Newfoundland (Fig. 1C; P. Gagnon, personal observations), and both are abundant at FC (as observed during other studies at this site). Once spread homogeneously across the 0.81-m^2^ wave tank experimental area, the above noted quantities of covering materials matched the average abundance and distribution of same covering materials at FC. Kelp, although also commonly used as cover, was not included because most pieces were too light and inevitably transported outside of the experimental area in the presence of waves (as determined during preliminary trials). We spread covering materials homogeneously over the experimental area to give sea urchins even chances of coming across materials.

We created the six UVR-based treatments (SUN, SUL, SUI, LUN, LUL, LUI; Table 1) by installing over the experimental area, a wooden cover equipped with four light bulbs (Eco Terra Repti 6 Glow, Desert) emitting white light and UVA/UVB radiation downwards (Fig. 2C). Opaque rigid blackout sheets were fitted above the rest of the wave tank Figure 2C). The cover and blackout sheets blocked external light input to the experimental area while not interfering with wave action. The four light bulbs were off for the six dark light treatments (SDN, SDL, SDI, LDN, LDL, LDI; Table 1), and on for the six white light treatments (SWN, SWL, SWI, LWN, LWL, LWI; Table 1) and six UVR + white light treatments (SUN, SUL, SUI, LUN, LUL, LUI; Table 1). A transparent UVA&B blocking sheet (ACRYLITE® UV filtering OP3, colourless) was placed under the cover to filter out UVR for the six white light treatments. The wave generator was off for the six experimental treatments without waves (SDN, SWN, SUN, LDN, LWN, LUN; Table 1) and operated at a frequency of 15 complete back-and-forth wave cycles per minute for all the 0.1 and 0.2 m s^−1^ wave velocity treatments (Table 1). These frequency and velocities are consistent with those in other studies examining various aspects of marine invertebrate behaviour, including *S. droebachiensis* [8, 40]. In the present study we chose 0.2 m s^−1^ as the highest wave velocity based on preliminary trials and other studies indicating a much-reduced mobility of green sea urchin at velocities beyond 0.3 m s^−1^ [6, 8, 42]. The 0.1 and 0.2 m s^−1^ wave velocities were created by initially filling the wave tank to 38 and 40 cm water depths, respectively.

Each trial lasted 40 min to give sea urchins enough time to cover, as determined through preliminary trials that showed covering occurred within the first 40 min of exposure to wave action in the wave tank. A large aluminum frame sitting on the sides of the tank (Fig. 2A) held a digital camera (PowerShot D30; Canon) used to take images of the experimental area at *t* = 0, 5, 10, 20, 30, and 40 min after the start of each trial. These images were supplemented by close-up images of the sea urchins to acquire additional information for validation purposes. Each trial began with the placement of five previously unused sea urchins on the centre tile, at ~10 cm between each other, to reduce the likelihood of interaction. The wooden cover with light bulbs was then placed over the experimental area, and the bulbs and wave generator were turned on as required by the experimental treatment (Table 1; Fig. 2C). At the end of each trial, we measured sea urchin t.d., wet weight, and weight of covering material (separated by material type) on their body surface. Sex was not considered in the present study to best capture covering trends in natural populations. Each treatment was replicated eight times (*n* = 8) from 9 to 24 July 2016. We ran one replicate of each treatment over two consecutive days in randomized order.

### Field survey

To characterize natural spatial and temporal variation in green sea urchin covering, while testing the generality of the results from the wave tank experiment, we tracked changes over three months in the type and quantity of covering materials used by *S. droebachiensis* at three depths in Flatrock Cove. In early May 2016, eye bolts were drilled to the seabed to mark the two extremities of one 30-m long transect at each depth: shallow (3 m), intermediate (5 m), and deep (9 m). These depths were chosen because they presented different habitats with different types and abundances of covering material to green sea urchins. The shallow habitat flanked the lower edge of a kelp bed where organic (including kelp and other seaweeds) and inorganic debris were noticeably more abundant than at the two other depths. The intermediate and deep habitats were both located in a sea urchin barren with virtually no fleshy seaweeds, except a few scattered plants of the brown seaweed *Desmarestia viridis* and patches of the kelp *Agarum clathratum*. Sea urchins in these two habitats were exposed to much lower (~75% less) hydrodynamic forces, and to a darker (~50% less) light environment (more so in the deepest habitat), than in the shallow habitat. On 31 May, 16 June, 10 July, 8 August, and 1 September 2016, divers attached a transect line at each depth, and filmed each side of the transect with a submersible video camera system (Sony HDV 1080i/MiniDV with an Amphibico Endeavor housing) propelled at a fixed distance (1.5 m) above the seabed. Twenty (20) quality images, each covering 0.5 × 0.25 m of seabed, were subsequently generated from each video transect and analyzed for covering.

### Image analysis

We analyzed images from the wave tank experiment and field survey with ImageJ (64-bit Java 1.8.0_172 [43]). For the wave tank experiment, we used the length of one tile (30 cm) to set the scale on each image. Each sea urchin’s displacement was determined by measuring and adding the linear distance it moved from one image (taken by the suspended camera) to the next. We used the close-up images taken at the end of each trial and ImageJ to calculate, for each sea urchin, the surface area of 1) the aboral side of the test [the only part of the test visible on each image given the camera’s angle], and 2) surface area of each type of covering material at the surface of the test. From these measurements we calculated, for each sea urchin, the proportion of the surface area of the test that was covered in each type of covering material (mussel shell pieces or rhodolith fragments). For the field images, sea urchins were assigned a value of 1 (presence of covering) or 0 (absence of covering) for analysis. For these images, we used the transect line’s divisions to set the scale on each image. Because sea urchins in the field varied in size, we also measured their t.d. and assigned them to either of the four following body size classes: [1- 2], [2–3], [3–4], and [4–5] cm. There were also more covering material types available to sea urchins in the field. For simplicity, we restricted the analysis to the four most abundant types noted: mussel shell pieces, rhodolith fragments, kelp, and “other”. The latter type included anything else than the first three types, i.e. mainly pebbles and other seaweeds. For analysis of material type, the presence or absence of covering was divided by each of the four material types.

### Statistical analysis

Wave Tank Experiment

#### Sea urchin displacement

A gamma GLM with an identity link [44] was used to characterize the relationship between sea urchin displacement (measured by distance moved in cm) and the proportion of aboral surface covered, while controlling for the factors wave velocity (null, low, and intermediate), light regime (dark, white, and UVR light exposure), and sea urchin body size (small and large in t.d.). The analysis was applied to the raw data (*n* = 687, 8 replicates of 18 treatments with mainly 5 sea urchins per replicate [sometimes 4 as a few sea urchins were not considered when their body orientation made it impossible to accurately determine the amount of surface covered]).

#### Covering

To analyze the frequency of covering, a binomial GLM with a logit link [45, 46] and factors wave velocity (null, low, and intermediate wave velocity), light regime (dark, white, and UVR light exposure), sea urchin body size (small and large in t.d.), and covering material type (mussel shell pieces, rhodolith fragments) was used to examine their effects on the proportion of sea urchins that exhibited covering in general as well as for each material type. Raw data (proportion of a sea urchin’s aboral surface covered) was rescaled into binary data (1 = presence of covering, 0 = absence of covering) for this analysis (*n* = 288, 8 replicates of 18 treatments for each material type with data averaged over 4–5 sea urchins per replicate).

To analyze the intensity of covering, a gamma GLM with a log link [44] and the same factors was used to examine their effects on the proportion of aboral surface area covered. Raw data was used for this analysis (*n* = 203), which included only sea urchins that exhibited covering. No significant interactions were found between material type and other factors in preliminary analyses (Tables A1 and A2). Such interactions were therefore removed from both models discussed above to better partition variation among factors and interactions relevant to our questions.

Field Survey

#### Covering (overall)

To analyze the frequency of covering, a binomial GLM with a logit link and factors date (five sampling dates), depth (shallow, intermediate, and deep sampling depths), and sea urchin body size class ([1–2], [2–3], [3–4], [4–5] cm t.d.) was used to examine their effects on the proportion of sea urchins that exhibited covering. Raw binary data (1 = presence of covering, 0 = absence of covering) was used for this analysis (*n* = 12,467 [total number of sea urchins observed]). We did not analyze the intensity of covering in field sea urchins because of the exploratory nature of this aspect of our research.

#### Covering (material type)

To analyze the frequency of covering more finely, a binomial GLM with a logit link and factors date (five sampling dates), depth (shallow, intermediate, and deep sampling depths), sea urchin body size class ([1–2], [2–3], [3–4], [4–5] cm t.d.), and covering material type (mussel shell pieces, rhodolith fragments, kelp, and “other”) was used to examine their effects on the proportion of sea urchins that covered with each material type. Raw data (proportion of a sea urchin’s aboral surface covered) was rescaled into binary data (1 = presence of covering with a given material, 0 = absence of covering with a given material) for this analysis (*n* = 49,868 [total number of sea urchins observed divided into each material type]). Interactions among the factors date, depth, and body size were examined in the previous analysis (see Sect. “Covering (overall)”). Accordingly, only interactions including the factor covering material type were considered in the present analysis.

#### General aspects of the analyses

All statistical analyses were carried out in R 4.2.0.1 (The R foundation 2016 [47]) and a significance threshold of 5% (α = 0.05) was used for all analyses. Model assumptions were examined for homogeneity and normality of the residuals using residual vs fitted plots and quantile-quantile plots. Generalized linear models were used for all analyses to account for heterogeneity of the residuals in the raw data. We used Tukey HSD multiple comparisons tests to detect differences among levels in a factor for any factor that showed significant effects.

## Results

### Wave tank experiment

#### Sea urchin displacement

**Fig. 3:**
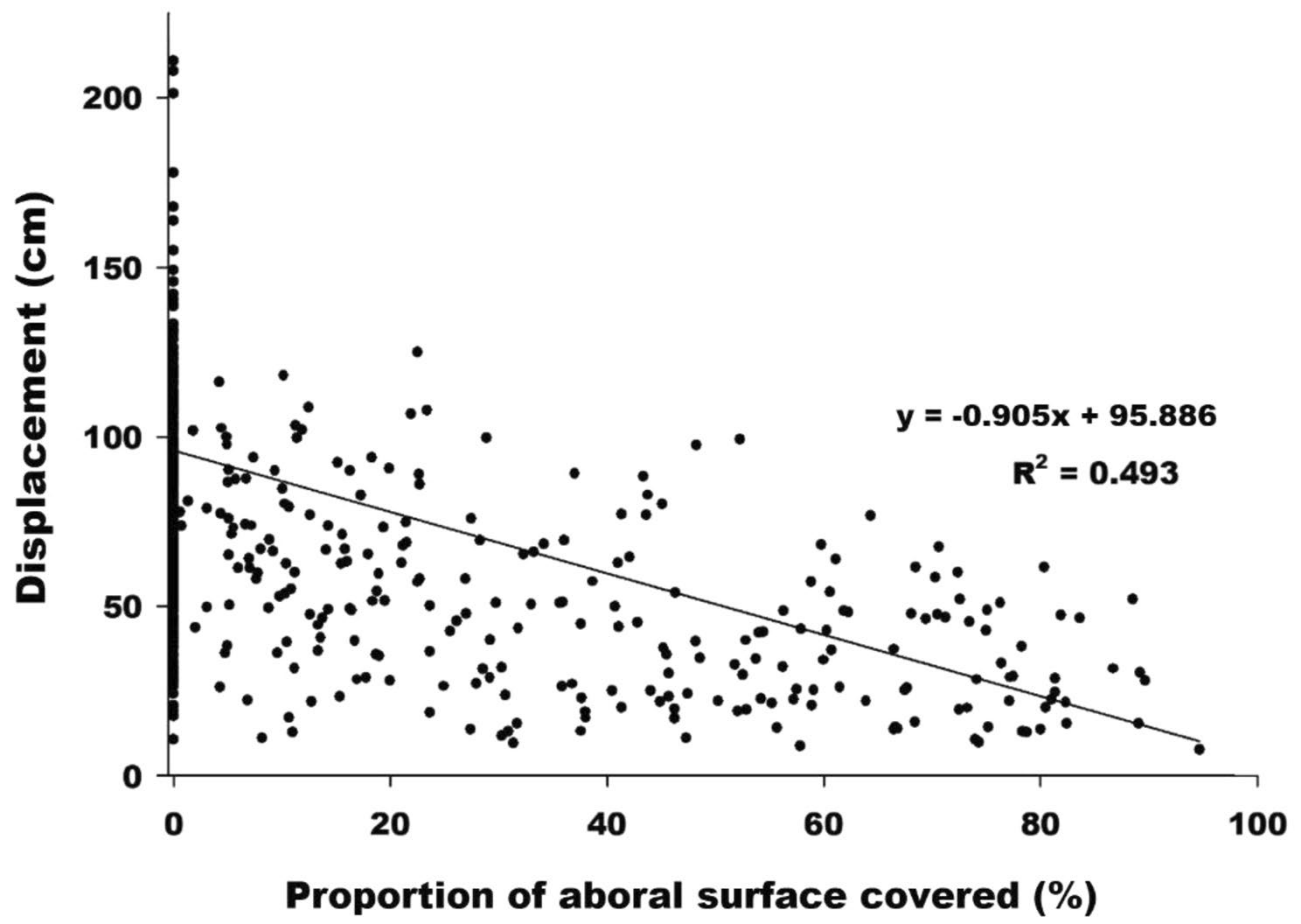
Relationship between green sea urchin (*Strongylocentrotus droebachiensis*) displacement and proportion of test (aboral surface) covered (both covering material types [mussel shell and rhodolith fragments] combined) over the 40-min trials in the wave tank experiment, with images taken at 0, 5, 10, 20, 30 and 40 min (see Sect. “Wave tank experiment” for a description of the experiment)

The gamma GLM analysis showed that the distance moved by sea urchins during the 40-min wave tank trials was inversely related to the proportion of test (aboral surface) covered, with ~9 cm decrease in distance for every 10% increase in test covered (*p* < 0.05) (Fig. 3).

#### Frequency of covering

**Fig. 4:**
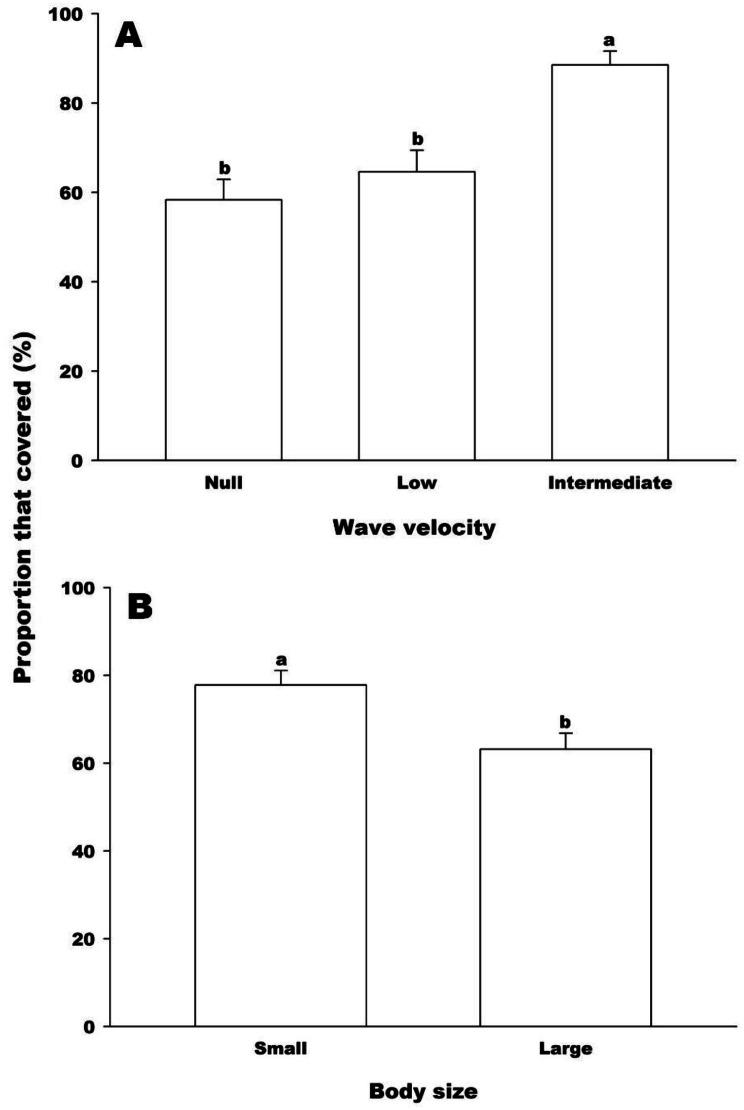
(**A**) Mean (+SE) proportion (frequency) of green sea urchins (*Strongylocentrotus droebachiensis*) that covered their aboral surface: (**A**) at each of the three wave velocities: null (0), low (0.1), and intermediate (0.2 m s^−1^), and (**B**) based on their body size: small (1 to 2 cm) and large (4 to 5 cm t.d.), in the wave tank experiment (both covering material types combined in both panels per Table 2). Bars not sharing the same letters differ statistically (LS means tests, *p* < 0.05; *n* = 48 for each wave velocity; *n* = 72 for each body size)

The binomial GLM analysis showed that both wave velocity and sea urchin body size significantly impacted the frequency of covering (proportion of urchins that covered) independently, with however no effects of light or material type (Table 2). Similar proportions of sea urchins covered their aboral surface in the absence of waves (58%) or presence of low wave velocity (65%) (*p* = 0.613), with a significantly higher majority (89%) of them doing so under intermediate wave velocity (*p* < 0.001) (Fig. 4A, Table A3). A significantly larger proportion of small sea urchins (78%) covered compared to the large ones (63%) (*p* < 0.003) (Fig. 4B, Table A4).

**Table 2:** Summary of analysis of deviance (ANODEV) (applied to rescaled data) for the binomial GLM examining the effects of light regime [light], wave velocity [velocity], sea urchin body size [size], and covering material type [material] on the frequency (proportion) of green sea urchins (*Strongylocentrotus droebachiensis*) that covered their aboral surface in the wave tank experiment (see Sect. “Wave tank experiment” for a description of the experiment)

Factor	df	Deviance	Residual df	Residual deviance	*p*(>|*z*|)
Light	2	5.6	285	343.8	0.060
Velocity	2	26.3	283	317.5	<0.001
Size	1	8.2	282	309.2	0.004
Material	1	2.3	281	306.8	0.127
Light X Velocity	4	7.1	277	299.7	0.129
Light X Size	2	3.1	275	296.6	0.209
Velocity X Size	2	1.5	273	295.1	0.468
Light X Velocity X Size	4	2.1	269	293.1	0.726

#### Intensity of covering

**Fig. 5:**
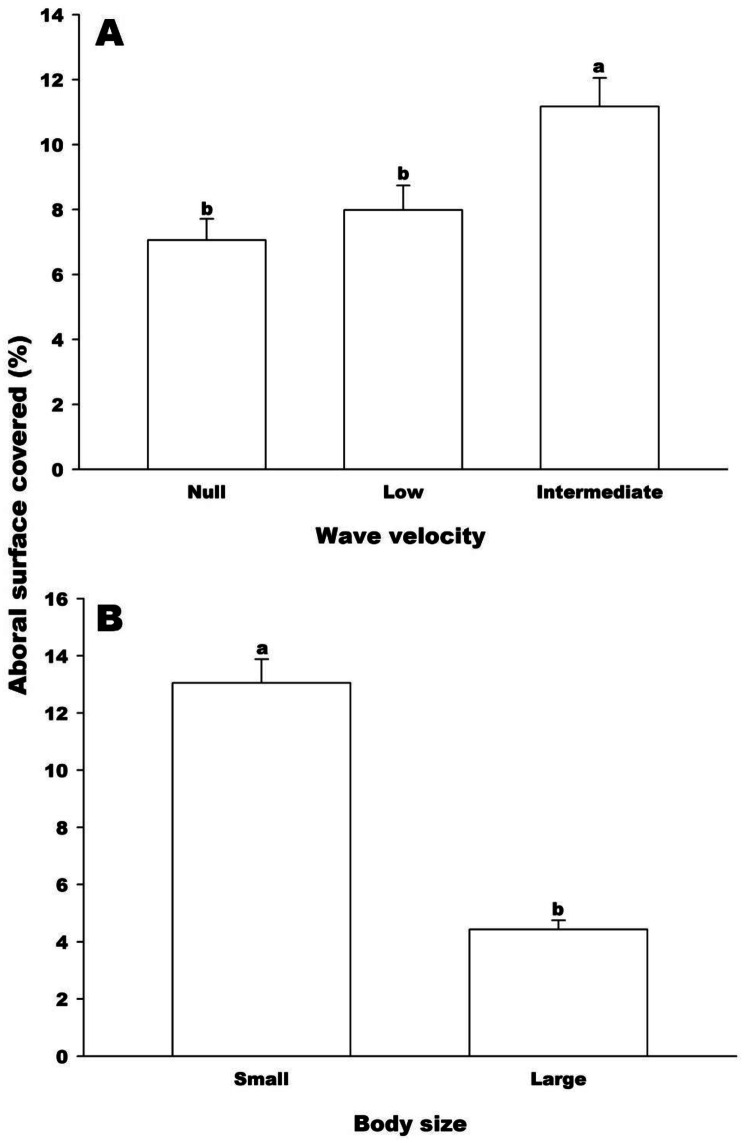
(**A**) Mean (+SE) proportion (intensity) of aboral surface covered in green sea urchins (*Strongylocentrotus droebachiensis*) that did cover: (**A**) at each of the three wave velocities: null (0), low (0.1), and intermediate (0.2 m s^−1^), and (**B**) based on their body size: small (1 to 2 cm) and large (4 to 5 cm t.d.), in the wave tank experiment (both covering material types combined in both panels per Table 3). Bars not sharing the same letters differ statistically (LS means test, *p* < 0.05; *n* = 56 to 85 for each wave velocity; *n* = 91 to 112 for each body size)

The gamma GLM analysis showed that both wave velocity and sea urchin body size significantly impacted the intensity of covering (proportion of aboral surface covered in those urchins that did cover) independently, with no effects of light and material type (Table 3). Sea urchins covered similar proportions of their aboral surface in the absence of waves (7%) or presence of low wave velocity (8%) (*p* = 0.625), with a significantly higher proportion of surface covered under intermediate wave velocity (11%) (*p* < 0.018) (Fig. 5A, Table A5). Small sea urchins covered significantly more of their surface (13%) than the large ones (4%) (*p* < 0.001) (Fig. 5B, Table A6).

**Table 3:** Summary of analysis of deviance (ANODEV) (applied to raw data) for the gamma GLM examining the effects of light regime [light], wave velocity [velocity], sea urchin body size [size], and covering material type [material] on the intensity of covering (proportion of aboral surface covered) in green sea urchins (*Strongylocentrotus droebachiensis*) that did cover in the wave tank experiment (see Sect. “Wave tank experiment” for a description of the experiment)

Factor	df	Deviance	Residual df	Residual deviance	*p*(>|*z*|)
Light	2	0.9	200	167.9	0.375
Velocity	2	5.5	198	162.5	0.003
Size	1	51.1	197	111.4	<0.001
Material	1	0.7	196	110.8	0.225
Light X Velocity	4	2.1	192	108.7	0.326
Light X Size	2	0.5	190	108.1	0.543
Velocity X Size	2	0.5	188	107.6	0.574
Light X Velocity X Size	4	1.8	184	106.6	0.658

### Field survey

The binomial GLM analysis of the frequency of covering showed that all two-way interactions between sampling date, sampling depth, and sea urchin body size were statistically significant, although no significant three-way interactions were (Table 4). The results of each two-way interactions are broken down accordingly in the next sections.

**Table 4:** Summary of analysis of deviance (ANODEV) (applied to binary data) for the binomial GLM examining the effects of sampling date [date], sampling depth [depth], and sea urchin body size [size] on the frequency (proportion) of green sea urchins (*Strongylocentrotus droebachiensis*) that covered their aboral surface in Flatrock Cove, from May to September 2016 (see Sect. “Field survey” for a description of the survey)

Factor	df	Deviance	Residual df	Residual deviance	*p*(>|*z*|)
Date	4	915.4	12462	16044	<0.001
Depth	2	658.7	12460	15386	<0.001
Size	3	117.8	12457	15268	<0.001
Date X Depth	8	134.9	12449	15133	<0.001
Date X Size	12	82.9	12437	15050	<0.001
Depth X Size	6	88.7	12431	14961	<0.001
Date X Depth X Size	24	34.5	12407	14927	0.075

#### Date × depth

**Fig. 6:**
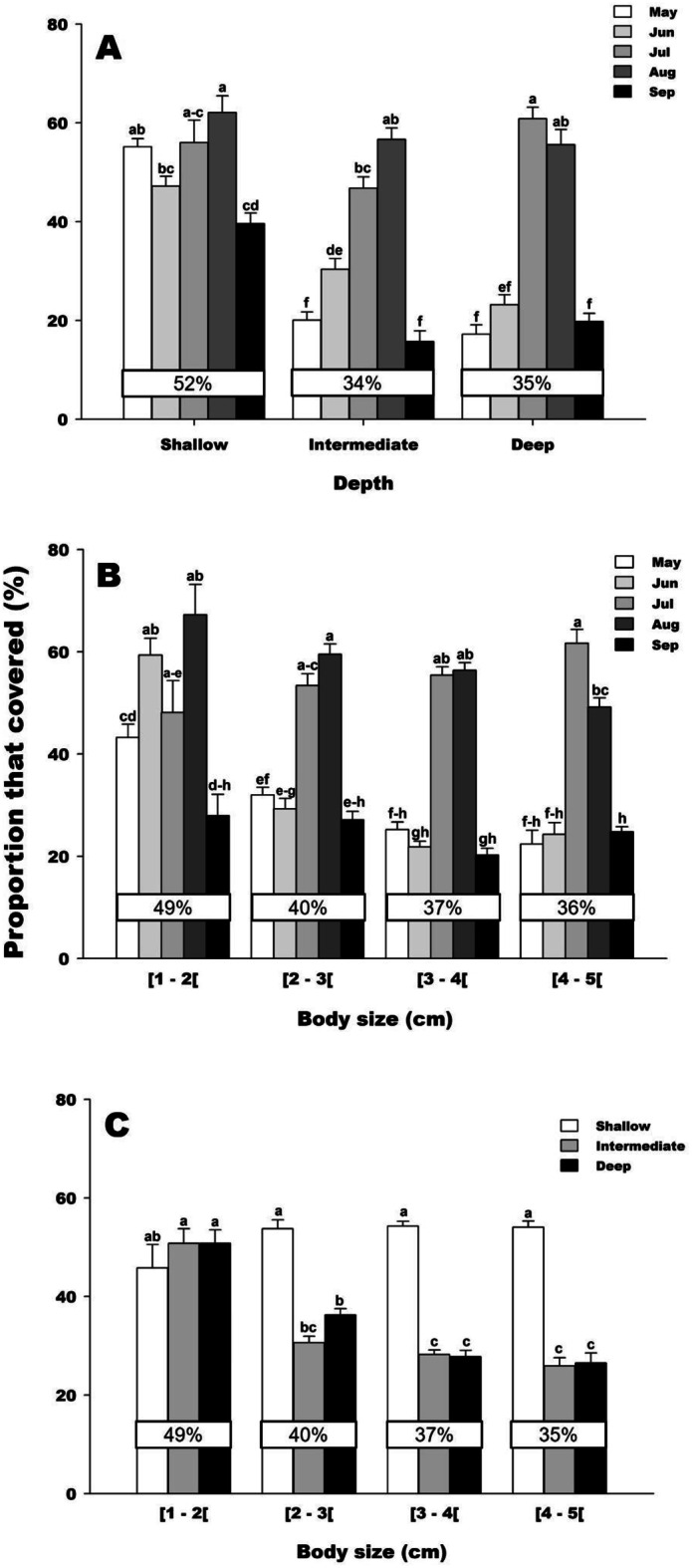
Mean (+SE) proportion (frequency) of green sea urchins (*Strongylocentrotus droebachiensis*) that covered their aboral surface (four covering material types combined) in the field survey, broken down per: (**A**) sampling date (31 May, 16 June, 10 July, 8 August, and 1 September, 2016) and sampling depth (shallow (3 m), intermediate (5 m), and Deep (9 m)), (**B**) sampling date (same five dates as above) and sea urchin body size (four size classes ranging from 1 to 5 cm in test diameter), and (**C**) sampling depth (same three depths as above) and sea urchin body size (same size classes as above). Boxed values are mean proportions across bars for each given combination of factors. Bars not sharing the same letters differ statistically (LS means tests, *p* < 0.05; *n* = 12,467)

The proportion of urchins that covered was generally highest (52%) in shallow (3 m) water throughout the survey, and similarly lowest at the intermediate depth (5 m, 34%) and in deep (9 m) water (35%) (Fig. 6A). In shallow water it peaked to 55–62% in May, July, and August (*p*  > 0.885, Table B1), while dropping significantly, by ~15%, from August to September (Fig. 6A, *p*  < 0.001, Table B1). At the intermediate depth (5 m), covering was lowest (20%) in May and increased significantly by ~ 10 to 17% per month over the next three months (Fig. 6A, *p* < 0.013, Table B1). It peaked in August at 57% and dropped markedly, back to the May proportion, in the last (September) month (Fig. 6A, *p* < 0.001, Table B1). The latter up and down seasonal trend was also observed in deep water, with an even more pronounced increase in covering of 38% from June (23%) to July (61%) (Fig. 6A, *p* < 0.001, Table B1). At all depths covering was systematically highest in July or August, ranging from 47% (July, intermediate) to 62% (August, shallow) (Fig. 6A).

#### Date × body size

The proportion of urchins that covered was generally highest (49%) in the smallest body size class (1 to <2 cm t.d.) throughout the survey, while decreasing slightly in each subsequently larger size classes (40% in [2–3], 37% in [3–4], and 36% in [4–5] cm t.d.) (Fig. 6B). Smallest urchins also exhibited the largest temporal variation in covering, with a month-to-month alternating up and down overall increase from May (43%) to August (67%), followed by an abrupt decline to 28% in September (Fig. 6B, *p* < 0.001, Table B2). The three larger size classes showed a sharper seasonal trend, with similar peaks (49–62%) in July and August and lows (20–32%) in May, June, and September (Fig. 6B). Like in the smallest urchins, covering in these larger size classes abruptly declined, to 20–27%, in the last month of the survey (Fig. 6B, *p* < 0.001, Table B2).

#### Depth × body size

The proportion of urchins that covered was generally highest (49%) in the smallest body size class (1 to <2 cm t.d.) across the three depths sampled, while decreasing slightly in each subsequently larger size classes (40% in [2–3], 37% in [3–4], and 35% in [4–5] cm t.d.) (Fig. 6C). Covering in the smallest urchins was similarly high (46–51%) across depths (Fig. 6C, *p*  > 0.999, Table B3). The three larger size classes showed a clear depth-related pattern, whereby covering was about two times higher (~54%) in shallow (3 m) water than at the intermediate depth (5 m, 26–31%) and in deep (9 m) water (27–36%) (Fig. 6C, *p* < 0.001, Table B3). In all size classes, covering was similarly highest in shallow water (Fig. 6C, *p*  > 0.852, Table B3).

### Covering material

Because interactions among the factors sampling date, sampling depth, and urchin body size were examined in the previous analysis, only interactions between each of these factors and covering material type were considered in the present analysis (Table 5). The results are broken down accordingly in the next sections.

**Table 5:** Summary of analysis of deviance (ANODEV) (applied to binary data) for the binomial GLM examining the effects of sampling date [date], sampling depth [depth], and sea urchin body size [size] on the frequency (proportion) of green sea urchins (*Strongylocentrotus droebachiensis*) that covered their aboral surface with each of the four material types [material] considered in the field survey (see Sect. “Field survey” for a description of the survey)

Factor	df	Deviance	Residual Df	Residual Deviance	*p*(>|*z*|)
Material	3	1013.7	49864	35153	<0.001
Date	4	755.1	49860	34398	<0.001
Depth	2	599.9	49858	33798	<0.001
Size	3	57.5	49855	33740	<0.001
Material X Date	12	1031.9	49843	32708	<0.001
Material X Depth	6	2637.2	49837	30071	<0.001
Material X Size	9	141.7	49828	29930	<0.001

#### Date × material

**Fig. 7:**
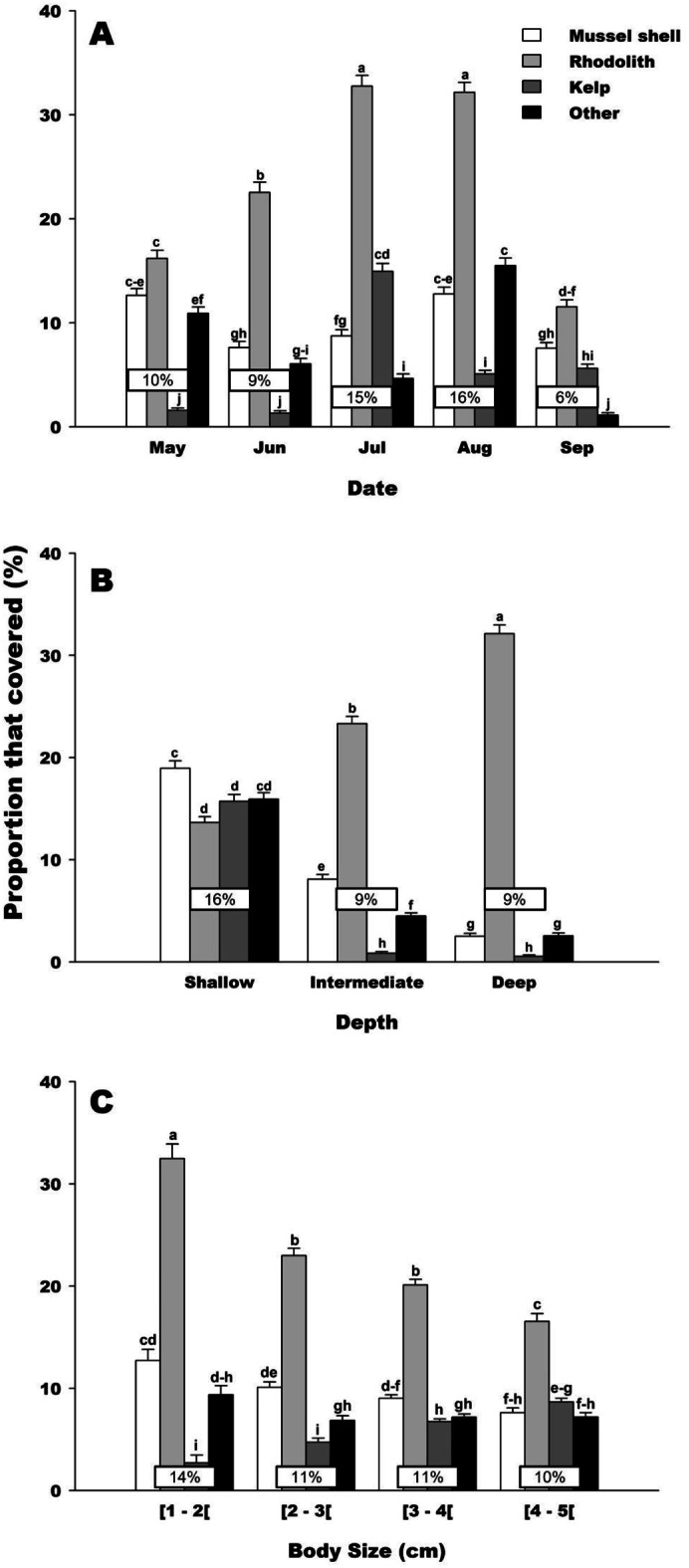
Mean (+SE) proportion of green sea urchins (*Strongylocentrotus droebachiensis*) covering with the four material types (mussel shell pieces, rhodolith fragments, kelp, and “other”) considered in the field survey, broken down per: (**A**) sampling date (31 May, 16 June, 10 July, 8 August, and 1 September, 2016), (**B**) sampling depth (shallow (3 m), intermediate (5 m), and deep (9 m)), and (**C**) sea urchin body size (four size classes ranging from 1 to 5 cm in test diameter). Bars not sharing the same letters differ statistically (LS means tests, *p* < 0.05; *n* = 49, 868)

The overall proportion of sea urchins that covered (regardless of material type) was generally highest in July (15%) and August (16%), followed by May (10%), June (9%), and September (6%) (Fig. 7A). In May, sea urchins primarily covered with rhodoliths (16%), mussel shells (13%), and “other” materials (11%), and to a much lesser extent with kelp (2%) (Fig. 7A, *p* < 0.001, Table B4). Rhodolith clearly was the dominant covering material throughout the remainder of the survey, being used ~2 to 4 times more frequently than any of the other materials (Fig. 7A, *p* < 0.001, Table B4). Kelp use peaked in July (15%), being at least twice more frequent than in any other months (Fig. 7A, *p* < 0.001, Table B4). Sea urchins covered with mussel shells primarily in May and August (~13%), and significantly less so in the other months (8–9%) (Fig. 7A, *p* < 0.001, Table B4).

#### Depth × material

The overall proportion of sea urchins that covered (regardless of material type) was generally highest in shallow water (16%), where it nearly doubled that at the intermediate depth and in deep water (both ~9%) (Fig. 7B). In shallow water, sea urchins covered predominantly with mussel shells (19%), and to a lesser extent (14–16%) with rhodolith or kelp (Fig. 7B, *p* < 0.041, Table B5). Rhodolith clearly was the dominant covering material used at the intermediate depth and in deep water, being used ~3 to 10 times more frequently than any of the other materials (Fig. 7B, *p* < 0.001, Table B5). Covering with mussel shell or other material both decreased significantly with increasing depth (Fig. 7B). Kelp use was an order of magnitude higher in shallow water than at the intermediate depth and in deep water, there being only marginal (~1%) (Fig. 7B, *p* < 0.001, Table B5). Rhodolith was the only material that sea urchins used systematically and at an increasing frequency with increasing depth (Fig. 7B).

#### Body size × material

The overall proportion of sea urchins that covered (regardless of material type) did not vary appreciably among the four body size classes, being only marginally (by ~3%) higher in the smallest ([1–2] cm t.d.) individuals (14%) compared to any of the three other (larger) size classes (10–11%) (Fig. 7C). Mussel shells and “other” materials were moderately (7–13%) used by all size classes. Kelp was the only material that sea urchins used at an increasing frequency with increasing body size, albeit also only relatively infrequently (27%) (Fig. 7C). Rhodolith was by far the most frequently used material (17-32%) across all size classes, and the only one used progressively less with increasing body size (Fig. 7C). The smallest proportion of sea urchins that covered with rhodoliths, i.e. 17% in the largest ([4–5] cm t.d) individuals, was significantly higher than the second next largest proportion of sea urchins that covered with any of the three other types of materials, i.e. 10% in the second smallest ([2–3] cm t.d.) individuals that covered with mussel shells (Fig. 7C, *p* < 0.001, Table B6).

## Discussion

Results of our multifactorial wave tank experiment with, and 3-mo survey of, green sea urchin, *Strongylocentrotus droebachiensis*, in a natural habitat in southeastern Newfoundland (Canada) were highly cohesive, refuting established paradigms, while providing new insights into the causes and consequences of covering in this pervasive marine herbivore. We propose that covering primarily serves a mechanical protection function.

### Wave tank experiment

#### Displacement

Our finding that green sea urchin displacement in the wave tank experiment decreased by 9 cm for every 10% increase in proportion of aboral surface covered (with mussel shell and rhodolith fragments) supported the prediction that displacement and covering are inversely related. “Uncovered” green sea urchins modulate aggregation and displacement in response to changes in sea temperature and wave action [6, 48], while favoring microhabitats that facilitate anchorage as wave velocity increases in the absence of food [8]. When facing physical barriers, uncovered green sea urchins can also alter movement patterns and reorient themselves towards areas with high food availability [49]. Collectively, these and other studies with other sea urchin species [e.g. 33, 35], suggest energetic trade-offs among essential functions such as aggregation, foraging, and displacement. Our finding reinforces this notion by showing that a natural biological process, namely covering, significantly reduces green sea urchin’s mobility.

#### UVR exposure

We showed that exposure to UV-enriched light did not alter sea urchin covering, contradicting our second prediction that the frequency and intensity of covering would be higher in the presence of UVR. Sharp and Gray [32] showed negative phototaxis to UVR in the sea urchins *Arbacia punctulata* and *Lytechinus variegatus*, with a stronger and more immediate behavioural response in the latter species including sheltering, fleeing, and covering. Our prediction was based on a few studies of *S. droebachiensis* suggesting that UVR can induce covering, however to a lesser extent than other competing factors such as urchin body size, exposure to waves, and algal whiplash [27, 31]. A possible explanation for the lack of green sea urchin response to UVR in our experiment could be that UVR irradiance was too low to induce covering. However, because UV light in coastal waters typically attenuates to only 10% within the first few meters [18], it seems unlikely that the amount of UVR shed on urchins in the wave tank, which contained only a few centimeters of water, would have been lower than the amount reaching sea urchins in natural habitats. Our other findings (see below) unequivocally demonstrate the strong influence of competing factors, which ultimately appears to override any potential, and at best weak, response of *S. droebachiensis* to UVR in natural habitats.

#### Wave velocity

Our results supported our prediction that the frequency and intensity of covering relate directly to wave velocity, while revealing a threshold value of ~0.1 m s^−1^ above which covering markedly increases. Dumont et al. [24] also found higher covering in *S. droebachiensis* at higher wave velocities similar to those in the present study. In a study of the spatial dynamics of the green sea urchin in food-depleted habitats, Frey and Gagnon [8] determined that shifts in wave velocity and population density in the order of 0.1 m s^−1^ and a few tens of individuals m^−2^, respectively, can elicit important changes in the way “uncovered” sea urchins disperse, cluster, and use seabed topography. They also pointed out that the strong inclination of uncovered sea urchins to reduce displacement, favor microhabitats that facilitate anchorage, and increasingly form 2-dimensional aggregations as wave velocity increases in the absence of food, is most likely a behavioural adaptation to mitigate hydrodynamic forces while reinforcing attachment to minimize dislodgement risk [8]. By ruling out the UVR-induced covering hypothesis, while showing that *S. droebachiensis* markedly increases covering above a threshold wave velocity and does so at the expense of reduced mobility (see above), our results support the notion that covering serves a complementary function to mitigation of hydrodynamic forces (see below).

#### Body size

We showed that small (1 to 2 cm t.d.) sea urchins covered systematically more (both in frequency and intensity) than large (4 to 5 cm t.d.) ones, therefore supporting our prediction that covering in *S. droebachiensis* is inversely related to body size. This finding corroborates similar trends in other studies of green sea urchin [24, 34] and *Paracentrotus lividus* [30]. As they grow, sea urchins, including *S. droebachiensis*, generally shift from a predominantly cryptic and sedentary behaviour to a largely mobile and active foraging modality [50–52]. Smaller green sea urchins tend to hide and seek shelter, which could be due to a greater sensitivity or vulnerability to environmental stressors, presumably alleviated in part by covering their body with various materials [24]. However, as noted above, our findings clearly indicate that covering also intensifies with increasing wave velocity, regardless of body size. Accordingly, and given the lack of interactive effects between body size and wave velocity on covering in any of our analyses, we propose that covering in *S. droebachiensis* serves primarily as a mechanical protection mechanism. This conclusion is further backed by (1) our demonstrated lack of preference between the two types of covering materials (mussel shell and rhodolith fragments) available (in equal proportions) for covering in the wave tank experiment, and (2) substantial use of kelp, a normal food item, as covering material at our natural study site (see below).

### Field survey

Results from our 3-mo survey of the green sea urchin population in Flatrock Cove (FC) strongly supported our three predictions that the frequency of covering would be (1) inversely related to depth, while (2) varying seasonally, with a generally (3) stronger response in small than large individuals. Because we found multiple interactive effects on covering between the factors sampling depth, sampling date, urchin body size, and type of covering material (see Results), we limit our discussion below to key findings as they relate to the dominant effects and their connection to findings from our wave tank experiment.

#### Covering frequency

We had based our first prediction above on known and anticipated effects of water turbulence on green sea urchin feeding, displacement, aggregation, and covering [see studies cited above, the present study], and the well-established principle that in shallow coastal areas, wave energy and its impact on benthic organisms attenuates with depth according to a logarithmic function that varies with wave period [53]. Although we did not quantify hydrodynamic forces in FC, scuba divers involved in the survey (including both authors on the present study) confirmed that hydrodynamic forces decreased with increasing depth across the 3-to-9 m depth range studied, especially on those more windy and wavy days. Our finding of a threshold depth of ~5 m below which covering markedly decreases to steady low levels speaks highly to the wave tank results in that they both demonstrate the existence of water current-induced behavioural (covering) tipping points in *S. droebachiensis*.

Interestingly, we also showed that covering at all depths was generally highest in the smallest urchins (whose body size was similar to that of the small urchins in the wave tank experiment) throughout the survey, while systematically highest in July and August in the three larger body size classes. Collectively, these findings indicate a continuous, natural inclination to cover in small green sea urchins and the existence of a seasonal component to covering in larger individuals. Frey and Gagnon [6] studied the impacts of thermal and hydrodynamic environmental variability on individual and aggregative feeding in *S. droebachiensis*. In conformity with the metabolic theory of ecology (MTE), they showed that individual feeding during early summer (June-July) obeyed a non-linear, size- and temperature-dependent relationship: feeding in large sea urchins was consistently highest and positively correlated with temperature < 12 °C and dropped within and above the 12–15 °C tipping range. This relationship was also more apparent in large than small urchins [6]. Sea temperature in coastal Newfoundland, including at FC, generally increases by 10–15 °C between June (~1–2°C) and early August (~12–16°C) [54, 55]. A likely explanation for our above noted finding, therefore, could be that larger sea urchins became metabolically depressed at FC in July and August, when sea temperature paralleled the above noted feeding tipping points, triggering a greater covering response. It is unlikely that the latter response would have been triggered by higher hydrodynamic forces in both months since there were no appreciable changes in wave frequency or intensity at FC throughout the entire survey period.

The greater hydrodynamic forces observed at our shallowest depth may partially explain our finding that covering in the larger sea urchins was about two times higher there than at the two greater depths. The presence of a thick kelp forest there may however also have triggered another behavioural shift in *S. droebachiensis*, with ramifications into covering. Several studies showed that sea urchins, including *S. droebachiensis* and *Strongylocentrotus polyacanthus*, avoid tactile contact with agitated algal fronds, including kelp [42, 56]. Moreover, Dumont et al. [24] showed that covering in larger green sea urchins increases in the vicinity of macroalgae under strong wave velocity. Accordingly, we propose that the above noted pattern at FC was a consequence of the direct effects of water turbulence reducing mobility in *S. droebachiensis*, which in combination with algal whiplash intensified covering, as supported by our wave tank experiment (i.e. the more the sea urchins cover the less they displace or vice-versa, see above).

#### Covering material

We showed that of the four types of covering materials we examined: (1) rhodolith clearly was the dominant type used by green sea urchins at FC throughout the majority of the survey period, and also the only material used at an increasing frequency with increasing depth; (2) kelp use at the shallowest depth was an order of magnitude higher than at the greater depths and varied seasonally with a clear peak in July (middle of the survey); and (3) mussel shell use was inversely related to depth. These distinct patterns, along with the lack of preference between two of these types of covering materials (mussel shell and rhodolith fragments) available in equal proportions in the wave tank experiment, indicate that *S. droebachiensis* modulates covering according to availability of covering materials.

In Newfoundland, rhodoliths (long-lived, non-geniculate, red coralline algae that grow as free-living balls, branched twigs, or rosettes) and the beds (biological communities) they form are a dominant component of shallow and deep subtidal seascapes [57, 58]. This is the case at FC, where an extensive rhodolith bed fringes the lower edge of the sea urchin barrens (P. Gagnon, personal observations). Rhodoliths are vulnerable to wave-induced breakage [59], which commonly occurs at FC during the stormy fall and winter months. Breakage creates fragments that are further exported by waves and currents to shallower waters, where they eventually settle in cracks and crevices. This phenomenon provides a steady supply of rhodolith fragments, which can be picked up and used as covering material by *S. droebachiensis*, therefore maximizing availability for covering year-round.

As noted above, kelp was another dominant habitat component within the first three meters of water at FC throughout our survey. In contrast to the deeper, permanent rhodolith bed, the kelp forest at FC (and at many other locations along the coast of Newfoundland) is much more ephemeral due to the annual life cycle of the dominant kelp species (*Alaria esculenta*) making up the bed [6, 40, 55]. Like in other high-latitude kelp forests, kelp fragments (detritus) at FC are often produced and exported to greater depths [40, 60], where they are either consumed by marine organisms, including *S. droebachiensis*, or, as shown by the present study, used as covering material. In eastern Canada, including Newfoundland, destructive grazing of kelp by green sea urchin, which creates kelp fragments, typically peaks during the summer months [5, 61, 62]. Our observed peak use of kelp as covering material in July at FC coincided with peak availability in kelp fragments. Although we know less about the specific population dynamics of blue mussel, *Mytilus edulis* at FC, studies indicate significant mortality of up to 98% in individuals that settle in exposed locations [63], which FC is. That *M. edulis* shell use was inversely related to depth again is logical, considering that the species primarily recruits in the first few meters of water and death would result in the creation and dispersal of empty light shells with a greater accumulation in shallow than deeper waters.

## Conclusions

The present study establishes that covering in *S. droebachiensis* is: (1) predominantly controlled by hydrodynamic forces, with the existence of water current-induced covering tipping points; (2) ontogenetically determined, with a continuous inclination to cover in small individuals and a seasonal component to covering in larger individuals; (3) opportunistic, with multiple types of covering materials employed based on availability; and (4) functionally costly, as it significantly reduces mobility.

The question then arises as to why green sea urchin covers? Our findings largely ruled out the paradigm that light, including UVR, induces covering [24] or at least clearly marginalized it as a trigger or effector; sea urchins in our wave tank experiment covered just as much in the dark as they did under other light conditions containing various levels of white and UVR light. As discussed above, our results and those of other studies of the behavioural repertoire of *S. droebachiensis* [6, 8, 9], instead support the notion that covering serves a complementary function to mitigation of hydrodynamic forces, and in particular as a mechanical protection mechanism. Indeed, if *S. droebachiensis* only covered to reduce the risk of dislodgment, then it would not reduce, and even completely stop, displacement as it does when “uncovered” and exposed to the same wave velocities used in the present study [8].

Through pondering of possible causes, therefore, we propose that *S. droebachiensis* covers to primarily shield its body surface to mechanically protect its external organs against physical contact with moving debris of all sizes, from clay and sand particles, which could clog sensory/respiratory tube feet, to pebbles and cobbles, which could erode or break locomotory tube feet and spines or defensive pedicellariae and spines [64, 65]. The rate of contact with such debris would increase with increasing hydrodynamic forces, providing a logical explanation as to why, as we showed, green sea urchins, and more so the smaller and more fragile individuals, intensify covering as wave velocity increases. It appears, therefore, that the benefits of covering would outweigh the cost of our noted substantial reduction in displacement accompanying intensification of covering and resulting interference with other key biological functions such as aggregation and foraging.

The present study identifies threshold wave velocity and ocean depths that trigger shifts in green sea urchin covering. It distills current knowledge about this intriguing behaviour, providing the most comprehensive demonstration of its causes and consequences through elucidation of the individual and relative importance of alleged factors. Our combined experimental and observational approach provides novel information that can be used to feed predictive models of marine benthic community dynamics driven by this geographically pervasive [52] and economically and ecologically important marine grazer [e.g. [66–68]. This kind of information is critically needed in the Anthropocene where accelerating changes in sea state and temperature pose a major challenge to accurately predicting and managing shifts in coastal resources, including sea urchin populations [69–73]. Further studies should address the physiological efficiency, vulnerability, and resilience of *S. droebachiensis*’ external organs to physical contact with moving debris in the context of covering to validate or refute our overall conclusion that covering primarily serves a mechanical protection function.

## Electronic supplementary material

Below is the link to the electronic supplementary material.

Supplementary Material 1

## Data Availability

The datasets generated and/or analyzed during the current study are available from the corresponding author on reasonable request.
