# Supplementary material for: Drivers and functional consequences of covering in a pervasive marine grazer (green sea urchin, Strongylocentrotus droebachiensis)
Source: Front Zool. 2026 May 29;23:27. doi: 10.1186/s12983-026-00616-3 (PMC13386655; doi:10.1186/s12983-026-00616-3)
Supplement: Supplementary file 1 — Supplementary Material 1 [file 12983_2026_616_MOESM1_ESM.docx]

# Appendix A Wave Tank Experiment

Appendix A contains supplemental materials related to the wave tank experiment. It consists mainly of results of post hoc tests from the statistical analyses presented in the core sections (see Section 2.3 for a description of the experiment and Section 2.6 for a description of the statistical analyses).

**Table A1.** Summary of analysis of deviance (ANODEV) (applied to rescaled data) for the binomial GLM examining the effects of light regime [Light], wave velocity [Velocity], and sea urchin body size [Size] on the frequency (proportion) of green sea urchins (*Strongylocentrotus droebachiensis*) that covered their aboral surface in the wave tank experiment.

| Factor | *df* | Deviance | Residual  *df* | Residual  Deviance | *p*(>\|*z*\|) |
| --- | --- | --- | --- | --- | --- |
| Light | 2 | 5.6 | 285 | 343.8 | 0.060 |
| Velocity | 2 | 26.4 | 283 | 317.4 | <0.001 |
| Size | 1 | 8.3 | 282 | 309.2 | 0.004 |
| Material | 1 | 2.3 | 281 | 306.8 | 0.127 |
| Light X Velocity | 4 | 7.1 | 277 | 299.7 | 0.129 |
| Light X Size | 2 | 3.1 | 275 | 296.6 | 0.209 |
| Velocity X Size | 2 | 1.5 | 273 | 295.1 | 0.468 |
| Light X Material | 2 | 1.2 | 271 | 293.9 | 0.546 |
| Velocity X Material | 2 | 0.5 | 269 | 293.3 | 0.773 |
| Size X Material | 1 | 0.8 | 268 | 292.5 | 0.358 |
| Light X Velocity X Size | 4 | 2.1 | 264 | 290.5 | 0.734 |
| Light X Velocity X Material | 4 | 7.4 | 260 | 283.1 | 0.117 |
| Light X Size X Material | 2 | 2.3 | 258 | 280.8 | 0.319 |
| Velocity X Size X Material | 2 | 0.9 | 256 | 279.9 | 0.633 |
| Light X Velocity X Size X Material | 4 | 0.2 | 252 | 279.7 | 0.995 |

**Table A2.** Summary of analysis of deviance (ANODEV) (applied to raw data) for the gamma GLM examining the effects of light regime [Light], wave velocity [Velocity], and sea urchin body size [Size] on the intensity of covering (proportion of aboral surface covered) in green sea urchins (*Strongylocentrotus droebachiensis*) that did cover in the wave tank experiment.

| Factor | *df* | Deviance | Residual  *df* | Residual  Deviance | *p*(>\|*z*\|) |
| --- | --- | --- | --- | --- | --- |
| Light | 2 | 0.9 | 200 | 167.9 | 0.940 |
| Velocity | 2 | 5.5 | 198 | 162.5 | 5.896 |
| Size | 1 | 51.1 | 197 | 111.4 | 109.267 |
| Material | 1 | 0.7 | 196 | 110.8 | 1.413 |
| Light X Velocity | 4 | 2.1 | 192 | 108.7 | 1.115 |
| Light X Size | 2 | 0.5 | 190 | 108.1 | 0.584 |
| Velocity X Size | 2 | 0.5 | 188 | 107.6 | 0.531 |
| Light X Material | 2 | 0.1 | 186 | 107.5 | 0.116 |
| Velocity X Material | 2 | 1.5 | 184 | 106.1 | 1.589 |
| Size X Material | 1 | 0.4 | 183 | 105.7 | 0.797 |
| Light X Velocity X Size | 4 | 0.9 | 179 | 104.7 | 0.520 |
| Light X Velocity X Material | 4 | 0.8 | 175 | 103.9 | 0.429 |
| Light X Size X Material | 2 | 0.9 | 173 | 102.9 | 0.978 |
| Velocity X Size X Material | 2 | 0.5 | 171 | 102.5 | 0.507 |
| Light X Velocity X Size X Material | 4 | 2.2 | 167 | 100.3 | 1.188 |

**Table A3.** Tukey tests on the differences in frequency of covering in green sea urchin (*Strongylocentrotus droebachiensis*) between wave velocity levels used in the wave tank experiment.

| Comparison | Difference (%) | Std. Error | *df* | z-ratio | *p*(>*z*) |
| --- | --- | --- | --- | --- | --- |
| Null - Low | -6.3 | 6.62 | Inf | –0.25 | 0.613 |
| Null - Intermediate | -30.2 | 5.52 | Inf | –3.75 | <0.001 |
| Low - Intermediate | -23.9 | 5.70 | Inf | –3.12 | <0.001 |

**Table A4.** Tukey tests on the differences in frequency of covering in green sea urchin (*Strongylocentrotus droebachiensis*) between body size classes used in the wave tank experiment.

| Comparison | Difference (%) | Std. Error | *df* | z-ratio | *p*(>*z*) |
| --- | --- | --- | --- | --- | --- |
| Small - Large | 14.6 | 4.87 | Inf | -2.994 | 0.003 |

**Table A5.** Tukey tests on the differences in intensity of covering in green sea urchins (*Strongylocentrotus droebachiensis*) between wave velocity treatments used in the wave tank experiment.

| Comparison | Difference (%) | Std. Error | *df* | t-ratio | *p*(>*z*) |
| --- | --- | --- | --- | --- | --- |
| Null - Low | -0.9 | 1.00 | 184 | -0.926 | 0.625 |
| Null - Intermediate | -4.1 | 1.10 | 184 | -3.743 | <0.001 |
| Low - Intermediate | -3.2 | 1.16 | 184 | -2.739 | 0.018 |

**Table A6.** Tukey tests on the differences in intensity of covering in green sea urchins (*Strongylocentrotus droebachiensis*) between body size classes used in the wave tank experiment.

| Comparison | Difference (%) | Std. Error | *df* | t-ratio | *p*(>*z*) |
| --- | --- | --- | --- | --- | --- |
| Small - Large | 8.6 | 0.889 | 184 | -9.685 | <0.001 |

# Appendix B Field Survey

Appendix B contains supplemental materials related to the field survey. It consists mainly of results of post hoc tests from the statistical analyses presented in the core sections (see Section 2.4 for a description of the field survey and Section 2.6 for a description of statistical analyses).

**Table B1.** Tukey tests on the differences in frequency of covering in green sea urchin (*Strongylocentrotus droebachiensis*) across sampling dates and sampling depths.

| Comparison | Difference | Std. Error | *df* | z-ratio | *p*(>*z*) |
| --- | --- | --- | --- | --- | --- |
| (05/31/2016 Deep) - (06/16/2016 Deep) | –0.061 | 0.027 | Inf | –2.270 | 0.613 |
| (05/31/2016 Deep) - (07/10/2016 Deep) | –0.436 | 0.030 | Inf | –14.699 | <0.001 |
| (05/31/2016 Deep) - (08/08/2016 Deep) | –0.382 | 0.036 | Inf | –10.621 | <0.001 |
| (05/31/2016 Deep) - (09/01/2016 Deep) | –0.027 | 0.025 | Inf | –1.085 | 0.999 |
| (05/31/2016 Deep) - (05/31/2016 Kelp) | –0.379 | 0.026 | Inf | –14.718 | <0.001 |
| (05/31/2016 Deep) - (06/16/2016 Kelp) | –0.299 | 0.029 | Inf | –10.395 | <0.001 |
| (05/31/2016 Deep) - (07/10/2016 Kelp) | –0.389 | 0.049 | Inf | –8.009 | <0.001 |
| (05/31/2016 Deep) - (08/08/2016 Kelp) | –0.448 | 0.039 | Inf | –11.644 | <0.001 |
| (05/31/2016 Deep) - (09/01/2016 Kelp) | –0.223 | 0.030 | Inf | –7.533 | <0.001 |
| (05/31/2016 Deep) - (05/31/2016 Int) | –0.029 | 0.025 | Inf | –1.159 | 0.998 |
| (05/31/2016 Deep) - (06/16/2016 Int) | –0.131 | 0.029 | Inf | –4.607 | <0.001 |
| (05/31/2016 Deep) - (07/10/2016 Int) | –0.296 | 0.028 | Inf | –10.443 | <0.001 |
| (05/31/2016 Deep) - (08/08/2016 Int) | –0.394 | 0.029 | Inf | –13.736 | <0.001 |
| (05/31/2016 Deep) - (09/01/2016 Int) | 0.015 | 0.030 | Inf | 0.519 | 1.000 |
| (06/16/2016 Deep) - (07/10/2016 Deep) | –0.376 | 0.030 | Inf | –12.627 | <0.001 |
| (06/16/2016 Deep) - (08/08/2016 Deep) | –0.321 | 0.036 | Inf | –8.920 | <0.001 |
| (06/16/2016 Deep) - (09/01/2016 Deep) | 0.034 | 0.025 | Inf | 1.358 | 0.991 |
| (06/16/2016 Deep) - (05/31/2016 Kelp) | –0.318 | 0.026 | Inf | –12.323 | <0.001 |
| (06/16/2016 Deep) - (06/16/2016 Shallow) | –0.238 | 0.029 | Inf | –8.264 | <0.001 |
| (06/16/2016 Deep) - (07/10/2016 Shallow) | –0.328 | 0.049 | Inf | –6.752 | <0.001 |
| (06/16/2016 Deep) - (08/08/2016 Shallow) | –0.387 | 0.039 | Inf | –10.053 | <0.001 |
| (06/16/2016 Deep) - (09/01/2016 Shallow) | –0.162 | 0.030 | Inf | –5.469 | <0.001 |
| (06/16/2016 Deep) - (05/31/2016 Int) | 0.032 | 0.025 | Inf | 1.257 | 0.996 |
| (06/16/2016 Deep) - (06/16/2016 Int) | –0.071 | 0.029 | Inf | –2.470 | 0.462 |
| (06/16/2016 Deep) - (07/10/2016 Int) | –0.235 | 0.028 | Inf | –8.280 | <0.001 |
| (06/16/2016 Deep) - (08/08/2016 Int) | –0.334 | 0.029 | Inf | –11.595 | <0.001 |
| (06/16/2016 Deep) - (09/01/2016 Int) | 0.076 | 0.030 | Inf | 2.565 | 0.394 |
| (07/10/2016 Deep) - (08/08/2016 Deep) | 0.054 | 0.038 | Inf | 1.420 | 0.986 |
| (07/10/2016 Deep) - (09/01/2016 Deep) | 0.410 | 0.028 | Inf | 14.610 | <0.001 |
| (07/10/2016 Deep) - (05/31/2016 Shallow) | 0.058 | 0.029 | Inf | 2.005 | 0.796 |
| (07/10/2016 Deep) - (06/16/2016 Shallow) | 0.138 | 0.032 | Inf | 4.360 | 0.001 |
| (07/10/2016 Deep) - (07/10/2016 Shallow) | 0.048 | 0.050 | Inf | 0.949 | 1.000 |
| (07/10/2016 Deep) - (08/08/2016 Shallow) | –0.012 | 0.041 | Inf | –0.283 | 1.000 |
| (07/10/2016 Deep) - (09/01/2016 Shallow) | 0.214 | 0.032 | Inf | 6.605 | <0.001 |
| (07/10/2016 Deep) - (05/31/2016 Int) | 0.407 | 0.028 | Inf | 14.410 | <0.001 |
| (07/10/2016 Deep) - (06/16/2016 Int) | 0.305 | 0.031 | Inf | 9.743 | <0.001 |
| (07/10/2016 Deep) - (07/10/2016 Int) | 0.141 | 0.031 | Inf | 4.504 | 0.001 |
| (07/10/2016 Deep) - (08/08/2016 Int) | 0.042 | 0.032 | Inf | 1.332 | 0.992 |
| (07/10/2016 Deep) - (09/01/2016 Int) | 0.452 | 0.032 | Inf | 13.974 | <0.001 |
| (08/08/2016 Deep) - (09/01/2016 Deep) | 0.355 | 0.035 | Inf | 10.260 | <0.001 |
| (08/08/2016 Deep) - (05/31/2016 Shallow) | 0.003 | 0.035 | Inf | 0.098 | 1.000 |
| (08/08/2016 Deep) - (06/16/2016 Shallow) | 0.083 | 0.038 | Inf | 2.218 | 0.652 |
| (08/08/2016 Deep) - (07/10/2016 Shallow) | –0.007 | 0.054 | Inf | –0.123 | 1.000 |
| (08/08/2016 Deep) - (08/08/2016 Shallow) | –0.066 | 0.045 | Inf | –1.450 | 0.983 |
| (08/08/2016 Deep) - (09/01/2016 Shallow) | 0.159 | 0.038 | Inf | 4.169 | 0.003 |
| (08/08/2016 Deep) - (05/31/2016 Int) | 0.353 | 0.035 | Inf | 10.141 | <0.001 |
| (08/08/2016 Deep) - (06/16/2016 Int) | 0.251 | 0.037 | Inf | 6.719 | <0.001 |
| (08/08/2016 Deep) - (07/10/2016 Int) | 0.086 | 0.037 | Inf | 2.315 | 0.579 |
| (08/08/2016 Deep) - (08/08/2016 Int) | –0.012 | 0.038 | Inf | –0.329 | 1.000 |
| (08/08/2016 Deep) - (09/01/2016 Int) | 0.398 | 0.038 | Inf | 10.409 | <0.001 |
| (09/01/2016 Deep) - (05/31/2016 Shallow) | –0.352 | 0.024 | Inf | –14.785 | <0.001 |
| (09/01/2016 Deep) - (06/16/2016 Shallow) | –0.272 | 0.027 | Inf | –10.061 | <0.001 |
| (09/01/2016 Deep) - (07/10/2016 Shallow) | –0.362 | 0.048 | Inf | –7.610 | <0.001 |
| (09/01/2016 Deep) - (08/08/2016 Shallow) | –0.421 | 0.037 | Inf | –11.317 | <0.001 |
| (09/01/2016 Deep) - (09/01/2016 Shallow) | –0.196 | 0.028 | Inf | –7.019 | <0.001 |
| (09/01/2016 Deep) - (05/31/2016 Int) | –0.002 | 0.023 | Inf | –0.093 | 1.000 |
| (09/01/2016 Deep) - (06/16/2016 Int) | –0.104 | 0.027 | Inf | –3.900 | 0.008 |
| (09/01/2016 Deep) - (07/10/2016 Int) | –0.269 | 0.027 | Inf | –10.116 | <0.001 |
| (09/01/2016 Deep) - (08/08/2016 Int) | –0.368 | 0.027 | Inf | –13.615 | <0.001 |
| (09/01/2016 Deep) - (09/01/2016 Int) | 0.042 | 0.028 | Inf | 1.514 | 0.975 |
| (05/31/2016 Shallow) - (06/16/2016 Shallow) | 0.080 | 0.028 | Inf | 2.864 | 0.213 |
| (05/31/2016 Shallow) - (07/10/2016 Shallow) | –0.010 | 0.048 | Inf | –0.211 | 1.000 |
| (05/31/2016 Shallow) - (08/08/2016 Shallow) | –0.069 | 0.038 | Inf | –1.833 | 0.885 |
| (05/31/2016 Shallow) - (09/01/2016 Shallow) | 0.156 | 0.029 | Inf | 5.421 | <0.001 |
| (05/31/2016 Shallow) - (05/31/2016 Int) | 0.350 | 0.024 | Inf | 14.523 | <0.001 |
| (05/31/2016 Shallow) - (06/16/2016 Int) | 0.247 | 0.028 | Inf | 8.965 | <0.001 |
| (05/31/2016 Shallow) - (07/10/2016 Int) | 0.083 | 0.027 | Inf | 3.013 | 0.148 |
| (05/31/2016 Shallow) - (08/08/2016 Int) | –0.016 | 0.028 | Inf | –0.568 | 1.000 |
| (05/31/2016 Shallow) - (09/01/2016 Int) | 0.394 | 0.029 | Inf | 13.713 | <0.001 |
| (06/16/2016 Shallow) - (07/10/2016 Shallow) | –0.090 | 0.050 | Inf | –1.809 | 0.895 |
| (06/16/2016 Shallow) - (08/08/2016 Shallow) | –0.149 | 0.040 | Inf | –3.734 | 0.016 |
| (06/16/2016 Shallow) - (09/01/2016 Shallow) | 0.076 | 0.032 | Inf | 2.414 | 0.505 |
| (06/16/2016 Shallow) - (05/31/2016 Int) | 0.270 | 0.027 | Inf | 9.890 | <0.001 |
| (06/16/2016 Shallow) - (06/16/2016 Int) | 0.168 | 0.030 | Inf | 5.507 | <0.001 |
| (06/16/2016 Shallow) - (07/10/2016 Int) | 0.003 | 0.030 | Inf | 0.096 | 1.000 |
| (06/16/2016 Shallow) - (08/08/2016 Int) | –0.096 | 0.031 | Inf | –3.120 | 0.112 |
| (06/16/2016 Shallow) - (09/01/2016 Int) | 0.314 | 0.032 | Inf | 9.985 | <0.001 |
| (07/10/2016 Shallow) - (08/08/2016 Shallow) | –0.059 | 0.056 | Inf | –1.059 | 0.999 |
| (07/10/2016 Shallow) - (09/01/2016 Shallow) | 0.166 | 0.050 | Inf | 3.304 | 0.066 |
| (07/10/2016 Shallow) - (05/31/2016 Int) | 0.360 | 0.048 | Inf | 7.543 | <0.001 |
| (07/10/2016 Shallow) - (06/16/2016 Int) | 0.258 | 0.050 | Inf | 5.196 | <0.001 |
| (07/10/2016 Shallow) - (07/10/2016 Int) | 0.093 | 0.050 | Inf | 1.876 | 0.865 |
| (07/10/2016 Shallow) - (08/08/2016 Int) | –0.006 | 0.050 | Inf | –0.114 | 1.000 |
| (07/10/2016 Shallow) - (09/01/2016 Int) | 0.404 | 0.050 | Inf | 8.050 | <0.001 |
| (08/08/2016 Shallow) - (09/01/2016 Shallow) | 0.225 | 0.041 | Inf | 5.551 | <0.001 |
| (08/08/2016 Shallow) - (05/31/2016 Int) | 0.419 | 0.037 | Inf | 11.205 | <0.001 |
| (08/08/2016 Shallow) - (06/16/2016 Int) | 0.317 | 0.040 | Inf | 7.969 | <0.001 |
| (08/08/2016 Shallow) - (07/10/2016 Int) | 0.152 | 0.040 | Inf | 3.835 | 0.011 |
| (08/08/2016 Shallow) - (08/08/2016 Int) | 0.054 | 0.040 | Inf | 1.341 | 0.992 |
| (08/08/2016 Shallow) - (09/01/2016 Int) | 0.463 | 0.041 | Inf | 11.428 | <0.001 |
| (09/01/2016 Shallow) - (05/31/2016 Int) | 0.194 | 0.028 | Inf | 6.883 | <0.001 |
| (09/01/2016 Shallow) - (06/16/2016 Int) | 0.092 | 0.031 | Inf | 2.935 | 0.180 |
| (09/01/2016 Shallow) - (07/10/2016 Int) | –0.073 | 0.031 | Inf | –2.349 | 0.553 |
| (09/01/2016 Shallow) - (08/08/2016 Int) | –0.172 | 0.031 | Inf | –5.457 | <0.001 |
| (09/01/2016 Shallow) - (09/01/2016 Int) | 0.238 | 0.032 | Inf | 7.391 | <0.001 |
| (05/31/2016 Int) - (06/16/2016 Int) | –0.102 | 0.027 | Inf | –3.784 | 0.013 |
| (05/31/2016 Int) - (07/10/2016 Int) | –0.267 | 0.027 | Inf | –9.941 | <0.001 |
| (05/31/2016 Int) - (08/08/2016 Int) | –0.365 | 0.027 | Inf | –13.412 | <0.001 |
| (05/31/2016 Int) - (09/01/2016 Int) | 0.044 | 0.028 | Inf | 1.578 | 0.964 |
| (06/16/2016 Int) - (07/10/2016 Int) | –0.165 | 0.030 | Inf | –5.481 | <0.001 |
| (06/16/2016 Int) - (08/08/2016 Int) | –0.263 | 0.030 | Inf | –8.657 | <0.001 |
| (06/16/2016 Int) - (09/01/2016 Int) | 0.147 | 0.031 | Inf | 4.694 | <0.001 |
| (07/10/2016 Int) - (08/08/2016 Int) | –0.099 | 0.030 | Inf | –3.255 | 0.076 |
| (07/10/2016 Int) - (09/01/2016 Int) | 0.311 | 0.031 | Inf | 10.011 | <0.001 |
| (08/08/2016 Int) - (09/01/2016 Int) | 0.410 | 0.031 | Inf | 13.035 | <0.001 |

**Table B2.** Tukey tests on the differences in frequency of covering in green sea urchin (*Strongylocentrotus droebachiensis*) across sampling dates and urchin body size classes [A = [1-2[, B = [2-3[, C = [3-4[, D = [4‑5[ cm t.d.).

| Comparison | Difference | Std. Error | *df* | z-ratio | *p*(>*z*) |
| --- | --- | --- | --- | --- | --- |
| ( D 05/31/2016) - ( A 05/31/2016) | –0.210 | 0.036 | Inf | –5.892 | <0.001 |
| ( D 05/31/2016) - ( B 05/31/2016) | –0.097 | 0.031 | Inf | –3.155 | 0.157 |
| ( D 05/31/2016) - ( C 05/31/2016) | –0.029 | 0.030 | Inf | –0.974 | 1.000 |
| ( D 05/31/2016) - ( D 06/16/2016) | –0.018 | 0.037 | Inf | –0.482 | 1.000 |
| ( D 05/31/2016) - ( A 06/16/2016) | –0.368 | 0.043 | Inf | –8.624 | <0.001 |
| ( D 05/31/2016) - ( B 06/16/2016) | –0.068 | 0.033 | Inf | –2.034 | 0.889 |
| ( D 05/31/2016) - ( C 06/16/2016) | 0.007 | 0.030 | Inf | 0.249 | 1.000 |
| ( D 05/31/2016) - ( D 07/10/2016) | –0.392 | 0.038 | Inf | –10.352 | <0.001 |
| ( D 05/31/2016) - ( A 07/10/2016) | –0.257 | 0.068 | Inf | –3.782 | 0.022 |
| ( D 05/31/2016) - ( B 07/10/2016) | –0.310 | 0.034 | Inf | –9.197 | <0.001 |
| ( D 05/31/2016) - ( C 07/10/2016) | –0.329 | 0.032 | Inf | –10.423 | <0.001 |
| ( D 05/31/2016) - ( D 08/08/2016) | –0.267 | 0.033 | Inf | –8.205 | <0.001 |
| ( D 05/31/2016) - ( A 08/08/2016) | –0.447 | 0.065 | Inf | –6.869 | <0.001 |
| ( D 05/31/2016) - ( B 08/08/2016) | –0.370 | 0.034 | Inf | –10.940 | <0.001 |
| ( D 05/31/2016) - ( C 08/08/2016) | –0.340 | 0.030 | Inf | –11.165 | <0.001 |
| ( D 05/31/2016) - ( D 09/01/2016) | 0.023 | 0.031 | Inf | 0.734 | 1.000 |
| ( D 05/31/2016) - ( A 09/01/2016) | –0.056 | 0.049 | Inf | –1.139 | 1.000 |
| ( D 05/31/2016) - ( B 09/01/2016) | –0.048 | 0.031 | Inf | –1.518 | 0.994 |
| ( D 05/31/2016) - ( C 09/01/2016) | –0.024 | 0.029 | Inf | –0.830 | 1.000 |
| ( A 05/31/2016) - ( B 0 5/31/2016) | 0.113 | 0.028 | Inf | 4.059 | 0.008 |
| ( A 05/31/2016) - ( C 0 5/31/2016) | 0.181 | 0.027 | Inf | 6.742 | <0.001 |
| ( A 05/31/2016) - ( D 06/16/2016) | 0.192 | 0.034 | Inf | 5.641 | <0.001 |
| ( A 05/31/2016) - ( A 0 6/16/2016) | –0.159 | 0.041 | Inf | –3.899 | 0.014 |
| ( A 05/31/2016) - ( B 0 6/16/2016) | 0.142 | 0.031 | Inf | 4.606 | 0.001 |
| ( A 05/31/2016) - ( C 0 6/16/2016) | 0.217 | 0.027 | Inf | 8.110 | <0.001 |
| ( A 05/31/2016) - ( D 07/10/2016) | –0.182 | 0.036 | Inf | –5.118 | <0.001 |
| ( A 05/31/2016) - ( A 0 7/10/2016) | –0.047 | 0.067 | Inf | –0.701 | 1.000 |
| ( A 05/31/2016) - ( B 0 7/10/2016) | –0.101 | 0.031 | Inf | –3.232 | 0.128 |
| ( A 05/31/2016) - ( C 0 7/10/2016) | –0.119 | 0.029 | Inf | –4.139 | 0.006 |
| ( A 05/31/2016) - ( D 08/08/2016) | –0.058 | 0.030 | Inf | –1.929 | 0.929 |
| ( A 05/31/2016) - ( A 0 8/08/2016) | –0.238 | 0.064 | Inf | –3.723 | 0.027 |
| ( A 05/31/2016) - ( B 0 8/08/2016) | –0.161 | 0.031 | Inf | –5.140 | <0.001 |
| ( A 05/31/2016) - ( C 0 8/08/2016) | –0.130 | 0.028 | Inf | –4.724 | <0.001 |
| ( A 05/31/2016) - ( D 09/01/2016) | 0.233 | 0.028 | Inf | 8.298 | <0.001 |
| ( A 05/31/2016) - ( A 0 9/01/2016) | 0.154 | 0.047 | Inf | 3.261 | 0.118 |
| ( A 05/31/2016) - ( B 0 9/01/2016) | 0.162 | 0.029 | Inf | 5.700 | <0.001 |
| ( A 05/31/2016) - ( C 0 9/01/2016) | 0.186 | 0.026 | Inf | 7.124 | <0.001 |
| ( B 05/31/2016) - ( C 0 5/31/2016) | 0.068 | 0.020 | Inf | 3.417 | 0.075 |
| ( B 05/31/2016) - ( D 06/16/2016) | 0.079 | 0.029 | Inf | 2.743 | 0.397 |
| ( B 05/31/2016) - ( A 0 6/16/2016) | –0.272 | 0.037 | Inf | –7.443 | <0.001 |
| ( B 05/31/2016) - ( B 0 6/16/2016) | 0.029 | 0.025 | Inf | 1.158 | 1.000 |
| ( B 05/31/2016) - ( C 0 6/16/2016) | 0.104 | 0.020 | Inf | 5.263 | <0.001 |
| ( B 05/31/2016) - ( D 07/10/2016) | -0.295 | 0.031 | Inf | –9.631 | <0.001 |
| ( B 05/31/2016) - ( A 0 7/10/2016) | -0.160 | 0.064 | Inf | –2.491 | 0.591 |
| ( B 05/31/2016) - ( B 0 7/10/2016) | -0.214 | 0.025 | Inf | –8.410 | <0.001 |
| ( B 05/31/2016) - ( C 0 7/10/2016) | -0.232 | 0.022 | Inf | –10.370 | <0.001 |
| ( B 05/31/2016) - ( D 08/08/2016) | -0.171 | 0.024 | Inf | –7.156 | <0.001 |
| ( B 05/31/2016) - ( A 0 8/08/2016) | -0.351 | 0.061 | Inf | –5.725 | <0.001 |
| ( B 05/31/2016) - ( B 0 8/08/2016) | -0.273 | 0.026 | Inf | –10.714 | <0.001 |
| ( B 05/31/2016) - ( C 0 8/08/2016) | -0.243 | 0.021 | Inf | –11.699 | <0.001 |
| ( B 05/31/2016) - ( D 09/01/2016) | 0.120 | 0.022 | Inf | 5.573 | <0.001 |
| ( B 05/31/2016) - ( A 0 9/01/2016) | 0.041 | 0.044 | Inf | 0.942 | 1.000 |
| ( B 05/31/2016) - ( B 0 9/01/2016) | 0.049 | 0.022 | Inf | 2.240 | 0.775 |
| ( B 05/31/2016) - ( C 0 9/01/2016) | 0.073 | 0.019 | Inf | 3.863 | 0.016 |
| ( C 05/31/2016) - ( D 06/16/2016) | 0.011 | 0.028 | Inf | 0.409 | 1.000 |
| ( C 05/31/2016) - ( A 0 6/16/2016) | -0.339 | 0.036 | Inf | –9.501 | <0.001 |
| ( C 05/31/2016) - ( B 0 6/16/2016) | -0.039 | 0.024 | Inf | –1.633 | 0.987 |
| ( C 05/31/2016) - ( C 0 6/16/2016) | 0.036 | 0.018 | Inf | 1.981 | 0.911 |
| ( C 05/31/2016) - ( D 07/10/2016) | -0.362 | 0.030 | Inf | –12.210 | <0.001 |
| ( C 05/31/2016) - ( A 0 7/10/2016) | -0.227 | 0.064 | Inf | –3.574 | 0.045 |
| ( C 05/31/2016) - ( B 0 7/10/2016) | -0.281 | 0.024 | Inf | –11.584 | <0.001 |
| ( C 05/31/2016) - ( C 0 7/10/2016) | -0.300 | 0.021 | Inf | –14.203 | <0.001 |
| ( C 05/31/2016) - ( D 08/08/2016) | -0.238 | 0.023 | Inf | –10.521 | <0.001 |
| ( C 05/31/2016) - ( A 0 8/08/2016) | -0.418 | 0.061 | Inf | –6.884 | <0.001 |
| ( C 05/31/2016) - ( B 0 8/08/2016) | -0.341 | 0.024 | Inf | –13.969 | <0.001 |
| ( C 05/31/2016) - ( C 0 8/08/2016) | -0.311 | 0.019 | Inf | –16.017 | <0.001 |
| ( C 05/31/2016) - ( D 09/01/2016) | 0.052 | 0.020 | Inf | 2.567 | 0.531 |
| ( C 05/31/2016) - ( A 0 9/01/2016) | -0.027 | 0.043 | Inf | –0.622 | 1.000 |
| ( C 05/31/2016) - ( B 0 9/01/2016) | -0.018 | 0.021 | Inf | –0.890 | 1.000 |
| ( C 05/31/2016) - ( C 0 9/01/2016) | 0.005 | 0.017 | Inf | 0.280 | 1.000 |
| ( D 06/16/2016) - ( A 06/16/2016) | -0.351 | 0.042 | Inf | –8.464 | <0.001 |
| ( D 06/16/2016) - ( B 06/16/2016) | -0.050 | 0.032 | Inf | –1.585 | 0.991 |
| ( D 06/16/2016) - ( C 06/16/2016) | 0.025 | 0.028 | Inf | 0.895 | 1.000 |
| ( D 06/16/2016) - ( D 07/10/2016) | -0.374 | 0.036 | Inf | –10.278 | <0.001 |
| ( D 06/16/2016) - ( A 07/10/2016) | -0.239 | 0.067 | Inf | –3.564 | 0.047 |
| ( D 06/16/2016) - ( B 07/10/2016) | -0.293 | 0.032 | Inf | –9.115 | <0.001 |
| ( D 06/16/2016) - ( C 07/10/2016) | -0.311 | 0.030 | Inf | –10.443 | <0.001 |
| ( D 06/16/2016) - ( D 08/08/2016) | -0.250 | 0.031 | Inf | –8.082 | <0.001 |
| ( D 06/16/2016) - ( A 08/08/2016) | -0.430 | 0.064 | Inf | –6.684 | <0.001 |
| ( D 06/16/2016) - ( B 08/08/2016) | -0.353 | 0.032 | Inf | –10.943 | <0.001 |
| ( D 06/16/2016) - ( C 08/08/2016) | -0.322 | 0.029 | Inf | –11.258 | <0.001 |
| ( D 06/16/2016) - ( D 09/01/2016) | 0.040 | 0.029 | Inf | 1.383 | 0.998 |
| ( D 06/16/2016) - ( A 09/01/2016) | -0.038 | 0.048 | Inf | –0.798 | 1.000 |
| ( D 06/16/2016) - ( B 09/01/2016) | -0.030 | 0.030 | Inf | –1.013 | 1.000 |
| ( D 06/16/2016) - ( C 09/01/2016) | -0.007 | 0.027 | Inf | –0.242 | 1.000 |
| ( A 06/16/2016) - ( B 0 6/16/2016) | 0.300 | 0.039 | Inf | 7.744 | <0.001 |
| ( A 06/16/2016) - ( C 0 6/16/2016) | 0.376 | 0.036 | Inf | 10.527 | <0.001 |
| ( A 06/16/2016) - ( D 07/10/2016) | -0.023 | 0.043 | Inf | –0.541 | 1.000 |
| ( A 06/16/2016) - ( A 0 7/10/2016) | 0.112 | 0.071 | Inf | 1.585 | 0.991 |
| ( A 06/16/2016) - ( B 0 7/10/2016) | 0.058 | 0.039 | Inf | 1.485 | 0.996 |
| ( A 06/16/2016) - ( C 0 7/10/2016) | 0.040 | 0.037 | Inf | 1.072 | 1.000 |
| ( A 06/16/2016) - ( D 08/08/2016) | 0.101 | 0.038 | Inf | 2.653 | 0.464 |
| ( A 06/16/2016) - ( A 0 8/08/2016) | -0.079 | 0.068 | Inf | –1.161 | 1.000 |
| ( A 06/16/2016) - ( B 0 8/08/2016) | -0.002 | 0.039 | Inf | –0.050 | 1.000 |
| ( A 06/16/2016) - ( C 0 8/08/2016) | 0.029 | 0.036 | Inf | 0.796 | 1.000 |
| ( A 06/16/2016) - ( D 09/01/2016) | 0.391 | 0.037 | Inf | 10.676 | <0.001 |
| ( A 06/16/2016) - ( A 0 9/01/2016) | 0.313 | 0.053 | Inf | 5.921 | <0.001 |
| ( A 06/16/2016) - ( B 0 9/01/2016) | 0.321 | 0.037 | Inf | 8.677 | <0.001 |
| ( A 06/16/2016) - ( C 0 9/01/2016) | 0.344 | 0.035 | Inf | 9.791 | <0.001 |
| ( B 06/16/2016) - ( C 0 6/16/2016) | 0.075 | 0.024 | Inf | 3.166 | 0.153 |
| ( B 06/16/2016) - ( D 07/10/2016) | -0.324 | 0.033 | Inf | –9.710 | <0.001 |
| ( B 06/16/2016) - ( A 0 7/10/2016) | -0.189 | 0.065 | Inf | –2.881 | 0.301 |
| ( B 06/16/2016) - ( B 0 7/10/2016) | -0.242 | 0.029 | Inf | –8.472 | <0.001 |
| ( B 06/16/2016) - ( C 0 7/10/2016) | -0.261 | 0.026 | Inf | –10.041 | <0.001 |
| ( B 06/16/2016) - ( D 08/08/2016) | -0.199 | 0.027 | Inf | –7.320 | <0.001 |
| ( B 06/16/2016) - ( A 0 8/08/2016) | -0.379 | 0.063 | Inf | –6.058 | <0.001 |
| ( B 06/16/2016) - ( B 0 8/08/2016) | -0.302 | 0.029 | Inf | –10.522 | <0.001 |
| ( B 06/16/2016) - ( C 0 8/08/2016) | -0.272 | 0.025 | Inf | –11.044 | <0.001 |
| ( B 06/16/2016) - ( D 09/01/2016) | 0.091 | 0.025 | Inf | 3.599 | 0.042 |
| ( B 06/16/2016) - ( A 0 9/01/2016) | 0.012 | 0.046 | Inf | 0.268 | 1.000 |
| ( B 06/16/2016) - ( B 0 9/01/2016) | 0.020 | 0.026 | Inf | 0.797 | 1.000 |
| ( B 06/16/2016) - ( C 0 9/01/2016) | 0.044 | 0.023 | Inf | 1.905 | 0.937 |
| ( C 06/16/2016) - ( D 07/10/2016) | -0.399 | 0.030 | Inf | –13.450 | <0.001 |
| ( C 06/16/2016) - ( A 0 7/10/2016) | -0.264 | 0.064 | Inf | –4.148 | 0.005 |
| ( C 06/16/2016) - ( B 0 7/10/2016) | -0.318 | 0.024 | Inf | –13.101 | <0.001 |
| ( C 06/16/2016) - ( C 0 7/10/2016) | -0.336 | 0.021 | Inf | –15.958 | <0.001 |
| ( C 06/16/2016) - ( D 08/08/2016) | -0.275 | 0.023 | Inf | –12.148 | <0.001 |
| ( C 06/16/2016) - ( A 0 8/08/2016) | -0.455 | 0.061 | Inf | –7.486 | <0.001 |
| ( C 06/16/2016) - ( B 0 8/08/2016) | -0.378 | 0.024 | Inf | –15.480 | <0.001 |
| ( C 06/16/2016) - ( C 0 8/08/2016) | -0.347 | 0.019 | Inf | –17.932 | <0.001 |
| ( C 06/16/2016) - ( D 09/01/2016) | 0.015 | 0.020 | Inf | 0.759 | 1.000 |
| ( C 06/16/2016) - ( A 0 9/01/2016) | -0.063 | 0.043 | Inf | –1.469 | 0.996 |
| ( C 06/16/2016) - ( B 0 9/01/2016) | -0.055 | 0.021 | Inf | –2.649 | 0.467 |
| ( C 06/16/2016) - ( C 0 9/01/2016) | -0.032 | 0.017 | Inf | –1.829 | 0.957 |
| ( D 07/10/2016) - ( A 07/10/2016) | 0.135 | 0.068 | Inf | 1.992 | 0.906 |
| ( D 07/10/2016) - ( B 07/10/2016) | 0.081 | 0.034 | Inf | 2.410 | 0.654 |
| ( D 07/10/2016) - ( C 07/10/2016) | 0.063 | 0.031 | Inf | 2.002 | 0.902 |
| ( D 07/10/2016) - ( D 08/08/2016) | 0.124 | 0.033 | Inf | 3.820 | 0.019 |
| ( D 07/10/2016) - ( A 08/08/2016) | -0.056 | 0.065 | Inf | –0.859 | 1.000 |
| ( D 07/10/2016) - ( B 08/08/2016) | 0.021 | 0.034 | Inf | 0.626 | 1.000 |
| ( D 07/10/2016) - ( C 08/08/2016) | 0.052 | 0.030 | Inf | 1.714 | 0.978 |
| ( D 07/10/2016) - ( D 09/01/2016) | 0.414 | 0.031 | Inf | 13.458 | <0.001 |
| ( D 07/10/2016) - ( A 09/01/2016) | 0.336 | 0.049 | Inf | 6.862 | <0.001 |
| ( D 07/10/2016) - ( B 09/01/2016) | 0.344 | 0.031 | Inf | 11.029 | <0.001 |
| ( D 07/10/2016) - ( C 09/01/2016) | 0.367 | 0.029 | Inf | 12.665 | <0.001 |
| ( A 07/10/2016) - ( B 0 7/10/2016) | -0.054 | 0.066 | Inf | –0.823 | 1.000 |
| ( A 07/10/2016) - ( C 0 7/10/2016) | -0.072 | 0.065 | Inf | –1.119 | 1.000 |
| ( A 07/10/2016) - ( D 08/08/2016) | -0.011 | 0.065 | Inf | –0.168 | 1.000 |
| ( A 07/10/2016) - ( A 0 8/08/2016) | -0.191 | 0.086 | Inf | –2.219 | 0.789 |
| ( A 07/10/2016) - ( B 0 8/08/2016) | -0.114 | 0.066 | Inf | –1.735 | 0.974 |
| ( A 07/10/2016) - ( C 0 8/08/2016) | -0.083 | 0.064 | Inf | –1.300 | 0.999 |
| ( A 07/10/2016) - ( D 09/01/2016) | 0.279 | 0.064 | Inf | 4.352 | 0.002 |
| ( A 07/10/2016) - ( A 0 9/01/2016) | 0.201 | 0.075 | Inf | 2.691 | 0.435 |
| ( A 07/10/2016) - ( B 0 9/01/2016) | 0.209 | 0.064 | Inf | 3.247 | 0.123 |
| ( A 07/10/2016) - ( C 0 9/01/2016) | 0.232 | 0.063 | Inf | 3.668 | 0.033 |
| ( B 07/10/2016) - ( C 0 7/10/2016) | -0.018 | 0.026 | Inf | –0.690 | 1.000 |
| ( B 07/10/2016) - ( D 08/08/2016) | 0.043 | 0.028 | Inf | 1.556 | 0.992 |
| ( B 07/10/2016) - ( A 0 8/08/2016) | -0.137 | 0.063 | Inf | –2.181 | 0.812 |
| ( B 07/10/2016) - ( B 0 8/08/2016) | -0.060 | 0.029 | Inf | –2.060 | 0.877 |
| ( B 07/10/2016) - ( C 0 8/08/2016) | -0.029 | 0.025 | Inf | –1.166 | 1.000 |
| ( B 07/10/2016) - ( D 09/01/2016) | 0.333 | 0.026 | Inf | 13.005 | <0.001 |
| ( B 07/10/2016) - ( A 0 9/01/2016) | 0.255 | 0.046 | Inf | 5.554 | <0.001 |
| ( B 07/10/2016) - ( B 0 9/01/2016) | 0.263 | 0.026 | Inf | 10.070 | <0.001 |
| ( B 07/10/2016) - ( C 0 9/01/2016) | 0.286 | 0.024 | Inf | 12.205 | <0.001 |
| ( C 07/10/2016) - ( D 08/08/2016) | 0.061 | 0.025 | Inf | 2.461 | 0.614 |
| ( C 07/10/2016) - ( A 0 8/08/2016) | -0.119 | 0.062 | Inf | –1.928 | 0.930 |
| ( C 07/10/2016) - ( B 0 8/08/2016) | -0.042 | 0.027 | Inf | –1.577 | 0.991 |
| ( C 07/10/2016) - ( C 0 8/08/2016) | -0.011 | 0.022 | Inf | –0.501 | 1.000 |
| ( C 07/10/2016) - ( D 09/01/2016) | 0.351 | 0.023 | Inf | 15.542 | <0.001 |
| ( C 07/10/2016) - ( A 0 9/01/2016) | 0.273 | 0.044 | Inf | 6.168 | <0.001 |
| ( C 07/10/2016) - ( B 0 9/01/2016) | 0.281 | 0.023 | Inf | 12.137 | <0.001 |
| ( C 07/10/2016) - ( C 0 9/01/2016) | 0.304 | 0.020 | Inf | 15.131 | <0.001 |
| ( D 08/08/2016) - ( A 08/08/2016) | -0.180 | 0.062 | Inf | –2.894 | 0.293 |
| ( D 08/08/2016) - ( B 08/08/2016) | -0.103 | 0.028 | Inf | –3.709 | 0.029 |
| ( D 08/08/2016) - ( C 08/08/2016) | -0.072 | 0.023 | Inf | –3.080 | 0.191 |
| ( D 08/08/2016) - ( D 09/01/2016) | 0.290 | 0.024 | Inf | 12.053 | <0.001 |
| ( D 08/08/2016) - ( A 09/01/2016) | 0.212 | 0.045 | Inf | 4.703 | 0.001 |
| ( D 08/08/2016) - ( B 09/01/2016) | 0.220 | 0.025 | Inf | 8.941 | <0.001 |
| ( D 08/08/2016) - ( C 09/01/2016) | 0.243 | 0.022 | Inf | 11.181 | <0.001 |
| ( A 08/08/2016) - ( B 0 8/08/2016) | 0.077 | 0.063 | Inf | 1.226 | 1.000 |
| ( A 08/08/2016) - ( C 0 8/08/2016) | 0.108 | 0.061 | Inf | 1.766 | 0.970 |
| ( A 08/08/2016) - ( D 09/01/2016) | 0.470 | 0.061 | Inf | 7.668 | <0.001 |
| ( A 08/08/2016) - ( A 0 9/01/2016) | 0.392 | 0.072 | Inf | 5.428 | <0.001 |
| ( A 08/08/2016) - ( B 0 9/01/2016) | 0.400 | 0.062 | Inf | 6.500 | <0.001 |
| ( A 08/08/2016) - ( C 0 9/01/2016) | 0.423 | 0.060 | Inf | 7.002 | <0.001 |
| ( B 08/08/2016) - ( C 0 8/08/2016) | 0.031 | 0.025 | Inf | 1.224 | 1.000 |
| ( B 08/08/2016) - ( D 09/01/2016) | 0.393 | 0.026 | Inf | 15.264 | <0.001 |
| ( B 08/08/2016) - ( A 0 9/01/2016) | 0.315 | 0.046 | Inf | 6.850 | <0.001 |
| ( B 08/08/2016) - ( B 0 9/01/2016) | 0.323 | 0.026 | Inf | 12.303 | <0.001 |
| ( B 08/08/2016) - ( C 0 9/01/2016) | 0.346 | 0.024 | Inf | 14.668 | <0.001 |
| ( C 08/08/2016) - ( D 09/01/2016) | 0.362 | 0.021 | Inf | 17.232 | <0.001 |
| ( C 08/08/2016) - ( A 0 9/01/2016) | 0.284 | 0.044 | Inf | 6.532 | <0.001 |
| ( C 08/08/2016) - ( B 0 9/01/2016) | 0.292 | 0.022 | Inf | 13.509 | <0.001 |
| ( C 08/08/2016) - ( C 0 9/01/2016) | 0.315 | 0.018 | Inf | 17.210 | <0.001 |
| ( D 09/01/2016) - ( A 09/01/2016) | -0.079 | 0.044 | Inf | –1.792 | 0.965 |
| ( D 09/01/2016) - ( B 09/01/2016) | -0.070 | 0.022 | Inf | –3.148 | 0.161 |
| ( D 09/01/2016) - ( C 09/01/2016) | -0.047 | 0.019 | Inf | –2.450 | 0.623 |
| ( A 09/01/2016) - ( B 0 9/01/2016) | 0.008 | 0.044 | Inf | 0.187 | 1.000 |
| ( A 09/01/2016) - ( C 0 9/01/2016) | 0.032 | 0.043 | Inf | 0.743 | 1.000 |
| ( B 09/01/2016) - ( C 0 9/01/2016) | 0.023 | 0.020 | Inf | 1.179 | 1.000 |

**Table B3.** Tukey tests on the differences in frequency of covering in green sea urchin (*Strongylocentrotus droebachiensis*) across sampling depths and urchin body size classes [A = [1-2[, B = [2-3[, C = [3-4[, D = [4-5[ cm t.d.).

| Comparison | Difference | Std. Error | *df* | z-ratio | *p*(>*z*) |
| --- | --- | --- | --- | --- | --- |
| D Deep - A Deep | –0.243 | 0.035 | Inf | –6.919 | <0.001 |
| D Deep - B Deep | –0.097 | 0.025 | Inf | –3.875 | 0.006 |
| D Deep - C Deep | –0.012 | 0.025 | Inf | –0.494 | 1.000 |
| D Deep - D Shallow | –0.275 | 0.026 | Inf | –10.700 | <0.001 |
| D Deep - A Shallow | –0.193 | 0.052 | Inf | –3.715 | 0.011 |
| D Deep - B Shallow | –0.273 | 0.027 | Inf | –9.971 | <0.001 |
| D Deep - C Shallow | –0.277 | 0.024 | Inf | –11.733 | <0.001 |
| D Deep - D Int | 0.006 | 0.027 | Inf | 0.221 | 1.000 |
| D Deep - A Int | –0.244 | 0.036 | Inf | –6.722 | <0.001 |
| D Deep - B Int | –0.042 | 0.025 | Inf | –1.695 | 0.871 |
| D Deep - C Int | –0.016 | 0.023 | Inf | –0.687 | 1.000 |
| A Deep - B Deep | 0.145 | 0.031 | Inf | 4.722 | <0.001 |
| A Deep - C Deep | 0.231 | 0.031 | Inf | 7.557 | <0.001 |
| A Deep - D Shallow | –0.032 | 0.031 | Inf | –1.024 | 0.997 |
| A Deep - A Shallow | 0.050 | 0.055 | Inf | 0.912 | 0.999 |
| A Deep - B Shallow | –0.030 | 0.033 | Inf | –0.929 | 0.999 |
| A Deep - C Shallow | –0.034 | 0.030 | Inf | –1.146 | 0.993 |
| A Deep - D Int | 0.249 | 0.033 | Inf | 7.645 | <0.001 |
| A Deep - A Int | –0.001 | 0.040 | Inf | –0.025 | 1.000 |
| A Deep - B Int | 0.201 | 0.031 | Inf | 6.584 | <0.001 |
| A Deep - C Int | 0.227 | 0.029 | Inf | 7.730 | <0.001 |
| B Deep - C Deep | 0.085 | 0.018 | Inf | 4.680 | <0.001 |
| B Deep - D Shallow | –0.177 | 0.019 | Inf | –9.141 | <0.001 |
| B Deep - A Shallow | –0.095 | 0.049 | Inf | –1.943 | 0.732 |
| B Deep - B Shallow | –0.176 | 0.022 | Inf | –8.128 | <0.001 |
| B Deep - C Shallow | –0.179 | 0.017 | Inf | –10.848 | <0.001 |
| B Deep - D Int | 0.103 | 0.021 | Inf | 4.824 | <0.001 |
| B Deep - A Int | –0.146 | 0.032 | Inf | –4.556 | <0.001 |
| B Deep - B Int | 0.055 | 0.018 | Inf | 3.048 | 0.095 |
| B Deep - C Int | 0.081 | 0.016 | Inf | 5.038 | <0.001 |
| C Deep - D Shallow | –0.263 | 0.019 | Inf | –13.857 | <0.001 |
| C Deep - A Shallow | –0.180 | 0.049 | Inf | –3.691 | 0.012 |
| C Deep - B Shallow | –0.261 | 0.021 | Inf | –12.299 | <0.001 |
| C Deep - C Shallow | –0.264 | 0.016 | Inf | –16.543 | <0.001 |
| C Deep - D Int | 0.018 | 0.021 | Inf | 0.869 | 0.999 |
| C Deep - A Int | –0.232 | 0.032 | Inf | –7.268 | <0.001 |
| C Deep - B Int | –0.030 | 0.018 | Inf | –1.681 | 0.877 |
| C Deep - C Int | –0.004 | 0.016 | Inf | –0.241 | 1.000 |
| D Shallow - A Shallow | 0.082 | 0.049 | Inf | 1.661 | 0.886 |
| D Shallow - B Shallow | 0.002 | 0.022 | Inf | 0.074 | 1.000 |
| D Shallow - C Shallow | –0.002 | 0.017 | Inf | –0.108 | 1.000 |
| D Shallow - D Int | 0.281 | 0.022 | Inf | 12.720 | <0.001 |
| D Shallow - A Int | 0.031 | 0.033 | Inf | 0.951 | 0.999 |
| D Shallow - B Int | 0.233 | 0.019 | Inf | 12.296 | <0.001 |
| D Shallow - C Int | 0.259 | 0.017 | Inf | 15.226 | <0.001 |
| A Shallow - B Shallow | –0.080 | 0.050 | Inf | –1.598 | 0.910 |
| A Shallow - C Shallow | –0.084 | 0.048 | Inf | –1.736 | 0.852 |
| A Shallow - D Int | 0.199 | 0.050 | Inf | 3.959 | 0.004 |
| A Shallow - A Int | –0.051 | 0.056 | Inf | –0.917 | 0.999 |
| A Shallow - B Int | 0.151 | 0.049 | Inf | 3.083 | 0.086 |
| A Shallow - C Int | 0.177 | 0.048 | Inf | 3.668 | 0.013 |
| B Shallow - C Shallow | –0.004 | 0.020 | Inf | –0.177 | 1.000 |
| B Shallow - D Int | 0.279 | 0.024 | Inf | 11.608 | <0.001 |
| B Shallow - A Int | 0.029 | 0.034 | Inf | 0.865 | 0.999 |
| B Shallow - B Int | 0.231 | 0.021 | Inf | 10.903 | <0.001 |
| B Shallow - C Int | 0.257 | 0.020 | Inf | 13.193 | <0.001 |
| C Shallow - D Int | 0.283 | 0.020 | Inf | 14.428 | <0.001 |
| C Shallow - A Int | 0.033 | 0.031 | Inf | 1.062 | 0.996 |
| C Shallow - B Int | 0.235 | 0.016 | Inf | 14.696 | <0.001 |
| C Shallow - C Int | 0.261 | 0.014 | Inf | 19.145 | <0.001 |
| D Int - A Int | –0.250 | 0.034 | Inf | –7.388 | <0.001 |
| D Int - B Int | –0.048 | 0.021 | Inf | –2.284 | 0.488 |
| D Int - C Int | –0.022 | 0.019 | Inf | –1.142 | 0.993 |
| A Int - B Int | 0.202 | 0.032 | Inf | 6.336 | <0.001 |
| A Int - C Int | 0.228 | 0.031 | Inf | 7.410 | <0.001 |
| B Int - C Int | 0.026 | 0.016 | Inf | 1.666 | 0.884 |

**Table B4.** Tukey tests on the differences in frequency of covering in green sea urchin (*Strongylocentrotus droebachiensis*) with the four different types of covering materials considered across sampling dates.

| Comparison | Difference | Std. Error | *df* | z-ratio | *p*(>*z*) |
| --- | --- | --- | --- | --- | --- |
| (kelp 05/31/2016) - (other 05/31/2016) | -0.093 | 0.006 | Inf | -14.464 | <0.001 |
| (kelp 05/31/2016) - (rhod 05/31/2016) | -0.146 | 0.008 | Inf | -18.026 | <0.001 |
| (kelp 05/31/2016) - (shell 05/31/2016) | -0.111 | 0.007 | Inf | -16.244 | <0.001 |
| (kelp 05/31/2016) - (kelp 06/16/2016) | 0.003 | 0.003 | Inf | 0.855 | 1.000 |
| (kelp 05/31/2016) - (other 06/16/2016) | -0.045 | 0.006 | Inf | -7.790 | <0.001 |
| (kelp 05/31/2016) - (rhod 06/16/2016) | -0.210 | 0.010 | Inf | -20.621 | <0.001 |
| (kelp 05/31/2016) - (shell 06/16/2016) | -0.060 | 0.006 | Inf | -9.560 | <0.001 |
| (kelp 05/31/2016) - (kelp 07/10/2016) | -0.134 | 0.008 | Inf | -17.465 | <0.001 |
| (kelp 05/31/2016) - (other 07/10/2016) | -0.031 | 0.005 | Inf | -6.194 | <0.001 |
| (kelp 05/31/2016) - (rhod 07/10/2016) | -0.312 | 0.011 | Inf | -29.526 | <0.001 |
| (kelp 05/31/2016) - (shell 07/10/2016) | -0.072 | 0.006 | Inf | -11.146 | <0.001 |
| (kelp 05/31/2016) - (kelp 08/08/2016) | -0.035 | 0.004 | Inf | -8.652 | <0.001 |
| (kelp 05/31/2016) - (other 08/08/2016) | -0.139 | 0.008 | Inf | -18.107 | <0.001 |
| (kelp 05/31/2016) - (rhod 08/08/2016) | -0.306 | 0.010 | Inf | -30.886 | <0.001 |
| (kelp 05/31/2016) - (shell 08/08/2016) | -0.112 | 0.007 | Inf | -16.067 | <0.001 |
| (kelp 05/31/2016) - (kelp 09/01/2016) | -0.040 | 0.004 | Inf | -8.994 | <0.001 |
| (kelp 05/31/2016) - (other 09/01/2016) | 0.004 | 0.003 | Inf | 1.486 | 0.996 |
| (kelp 05/31/2016) - (rhod 09/01/2016) | -0.100 | 0.007 | Inf | -14.280 | <0.001 |
| (kelp 05/31/2016) - (shell 09/01/2016) | -0.060 | 0.006 | Inf | -10.200 | <0.001 |
| (other 05/31/2016) - (rhod 05/31/2016) | -0.053 | 0.010 | Inf | -5.342 | <0.001 |
| (other 05/31/2016) - (shell 05/31/2016) | -0.017 | 0.009 | Inf | -1.961 | 0.918 |
| (other 05/31/2016) - (kelp 06/16/2016) | 0.096 | 0.006 | Inf | 14.886 | <0.001 |
| (other 05/31/2016) - (other 06/16/2016) | 0.049 | 0.008 | Inf | 6.240 | <0.001 |
| (other 05/31/2016) - (rhod 06/16/2016) | -0.116 | 0.012 | Inf | -10.002 | <0.001 |
| (other 05/31/2016) - (shell 06/16/2016) | 0.033 | 0.008 | Inf | 3.890 | 0.015 |
| (other 05/31/2016) - (kelp 07/10/2016) | -0.040 | 0.010 | Inf | -4.182 | 0.005 |
| (other 05/31/2016) - (other 07/10/2016) | 0.063 | 0.007 | Inf | 8.654 | <0.001 |
| (other 05/31/2016) - (rhod 07/10/2016) | -0.219 | 0.012 | Inf | -18.252 | <0.001 |
| (other 05/31/2016) - (shell 07/10/2016) | 0.022 | 0.009 | Inf | 2.552 | 0.543 |
| (other 05/31/2016) - (kelp 08/08/2016) | 0.058 | 0.007 | Inf | 8.344 | <0.001 |
| (other 05/31/2016) - (other 08/08/2016) | -0.046 | 0.009 | Inf | -5.058 | <0.001 |
| (other 05/31/2016) - (rhod 08/08/2016) | -0.212 | 0.011 | Inf | -18.648 | <0.001 |
| (other 05/31/2016) - (shell 08/08/2016) | -0.019 | 0.009 | Inf | -2.068 | 0.873 |
| (other 05/31/2016) - (kelp 09/01/2016) | 0.053 | 0.007 | Inf | 7.282 | <0.001 |
| (other 05/31/2016) - (other 09/01/2016) | 0.098 | 0.006 | Inf | 15.452 | <0.001 |
| (other 05/31/2016) - (rhod 09/01/2016) | -0.006 | 0.009 | Inf | -0.694 | 1.000 |
| (other 05/31/2016) - (shell 09/01/2016) | 0.034 | 0.008 | Inf | 4.132 | 0.006 |
| (rhod 05/31/2016) - (shell 05/31/2016) | 0.035 | 0.010 | Inf | 3.500 | 0.058 |
| (rhod 05/31/2016) - (kelp 06/16/2016) | 0.149 | 0.008 | Inf | 18.363 | <0.001 |
| (rhod 05/31/2016) - (other 06/16/2016) | 0.101 | 0.009 | Inf | 10.750 | <0.001 |
| (rhod 05/31/2016) - (rhod 06/16/2016) | -0.064 | 0.012 | Inf | -5.120 | <0.001 |
| (rhod 05/31/2016) - (shell 06/16/2016) | 0.086 | 0.010 | Inf | 8.757 | <0.001 |
| (rhod 05/31/2016) - (kelp 07/10/2016) | 0.012 | 0.011 | Inf | 1.140 | 1.000 |
| (rhod 05/31/2016) - (other 07/10/2016) | 0.115 | 0.009 | Inf | 12.858 | <0.001 |
| (rhod 05/31/2016) - (rhod 07/10/2016) | -0.166 | 0.013 | Inf | -13.062 | <0.001 |
| (rhod 05/31/2016) - (shell 07/10/2016) | 0.075 | 0.010 | Inf | 7.568 | <0.001 |
| (rhod 05/31/2016) - (kelp 08/08/2016) | 0.111 | 0.009 | Inf | 13.009 | <0.001 |
| (rhod 05/31/2016) - (other 08/08/2016) | 0.007 | 0.011 | Inf | 0.641 | 1.000 |
| (rhod 05/31/2016) - (rhod 08/08/2016) | -0.160 | 0.012 | Inf | -13.013 | <0.001 |
| (rhod 05/31/2016) - (shell 08/08/2016) | 0.034 | 0.010 | Inf | 3.349 | 0.092 |
| (rhod 05/31/2016) - (kelp 09/01/2016) | 0.106 | 0.009 | Inf | 12.053 | <0.001 |
| (rhod 05/31/2016) - (other 09/01/2016) | 0.151 | 0.008 | Inf | 18.674 | <0.001 |
| (rhod 05/31/2016) - (rhod 09/01/2016) | 0.047 | 0.010 | Inf | 4.622 | 0.001 |
| (rhod 05/31/2016) - (shell 09/01/2016) | 0.086 | 0.009 | Inf | 9.094 | <0.001 |
| (shell 05/31/2016) - (kelp 06/16/2016) | 0.113 | 0.007 | Inf | 16.645 | <0.001 |
| (shell 05/31/2016) - (other 06/16/2016) | 0.066 | 0.008 | Inf | 7.903 | <0.001 |
| (shell 05/31/2016) - (rhod 06/16/2016) | -0.099 | 0.012 | Inf | -8.364 | <0.001 |
| (shell 05/31/2016) - (shell 06/16/2016) | 0.050 | 0.008 | Inf | 5.933 | <0.001 |
| (shell 05/31/2016) - (kelp 07/10/2016) | -0.023 | 0.010 | Inf | -2.328 | 0.715 |
| (shell 05/31/2016) - (other 07/10/2016) | 0.080 | 0.008 | Inf | 10.217 | <0.001 |
| (shell 05/31/2016) - (rhod 07/10/2016) | -0.201 | 0.012 | Inf | -16.530 | <0.001 |
| (shell 05/31/2016) - (shell 07/10/2016) | 0.039 | 0.008 | Inf | 4.632 | 0.001 |
| (shell 05/31/2016) - (kelp 08/08/2016) | 0.076 | 0.007 | Inf | 10.332 | <0.001 |
| (shell 05/31/2016) - (other 08/08/2016) | -0.029 | 0.010 | Inf | -2.916 | 0.280 |
| (shell 05/31/2016) - (rhod 08/08/2016) | -0.195 | 0.012 | Inf | -16.819 | <0.001 |
| (shell 05/31/2016) - (shell 08/08/2016) | -0.001 | 0.009 | Inf | -0.134 | 1.000 |
| (shell 05/31/2016) - (kelp 09/01/2016) | 0.070 | 0.008 | Inf | 9.258 | <0.001 |
| (shell 05/31/2016) - (other 09/01/2016) | 0.115 | 0.007 | Inf | 17.024 | <0.001 |
| (shell 05/31/2016) - (rhod 09/01/2016) | 0.011 | 0.009 | Inf | 1.205 | 1.000 |
| (shell 05/31/2016) - (shell 09/01/2016) | 0.051 | 0.008 | Inf | 6.280 | <0.001 |
| (kelp 06/16/2016) - (other 06/16/2016) | -0.047 | 0.006 | Inf | -8.258 | <0.001 |
| (kelp 06/16/2016) - (rhod 06/16/2016) | -0.212 | 0.010 | Inf | -20.888 | <0.001 |
| (kelp 06/16/2016) - (shell 06/16/2016) | -0.063 | 0.006 | Inf | -9.987 | <0.001 |
| (kelp 06/16/2016) - (kelp 07/10/2016) | -0.136 | 0.008 | Inf | -17.915 | <0.001 |
| (kelp 06/16/2016) - (other 07/10/2016) | -0.033 | 0.005 | Inf | -6.736 | <0.001 |
| (kelp 06/16/2016) - (rhod 07/10/2016) | -0.314 | 0.011 | Inf | -29.786 | <0.001 |
| (kelp 06/16/2016) - (shell 07/10/2016) | -0.074 | 0.006 | Inf | -11.568 | <0.001 |
| (kelp 06/16/2016) - (kelp 08/08/2016) | -0.038 | 0.004 | Inf | -9.426 | <0.001 |
| (kelp 06/16/2016) - (other 08/08/2016) | -0.142 | 0.008 | Inf | -18.462 | <0.001 |
| (kelp 06/16/2016) - (rhod 08/08/2016) | -0.308 | 0.010 | Inf | -31.164 | <0.001 |
| (kelp 06/16/2016) - (shell 08/08/2016) | -0.114 | 0.007 | Inf | -16.459 | <0.001 |
| (kelp 06/16/2016) - (kelp 09/01/2016) | -0.043 | 0.004 | Inf | -9.660 | <0.001 |
| (kelp 06/16/2016) - (other 09/01/2016) | 0.002 | 0.003 | Inf | 0.619 | 1.000 |
| (kelp 06/16/2016) - (rhod 09/01/2016) | -0.102 | 0.007 | Inf | -14.670 | <0.001 |
| (kelp 06/16/2016) - (shell 09/01/2016) | -0.062 | 0.006 | Inf | -10.663 | <0.001 |
| (other 06/16/2016) - (rhod 06/16/2016) | -0.165 | 0.011 | Inf | -14.658 | <0.001 |
| (other 06/16/2016) - (shell 06/16/2016) | -0.016 | 0.008 | Inf | -1.970 | 0.915 |
| (other 06/16/2016) - (kelp 07/10/2016) | -0.089 | 0.009 | Inf | -9.671 | <0.001 |
| (other 06/16/2016) - (other 07/10/2016) | 0.014 | 0.007 | Inf | 2.126 | 0.844 |
| (other 06/16/2016) - (rhod 07/10/2016) | -0.267 | 0.012 | Inf | -23.014 | <0.001 |
| (other 06/16/2016) - (shell 07/10/2016) | -0.027 | 0.008 | Inf | -3.339 | 0.094 |
| (other 06/16/2016) - (kelp 08/08/2016) | 0.010 | 0.006 | Inf | 1.537 | 0.993 |
| (other 06/16/2016) - (other 08/08/2016) | -0.094 | 0.008 | Inf | -11.195 | <0.001 |
| (other 06/16/2016) - (rhod 08/08/2016) | -0.261 | 0.011 | Inf | -23.714 | <0.001 |
| (other 06/16/2016) - (shell 08/08/2016) | -0.067 | 0.008 | Inf | -7.930 | <0.001 |
| (other 06/16/2016) - (kelp 09/01/2016) | 0.004 | 0.007 | Inf | 0.655 | 1.000 |
| (other 06/16/2016) - (other 09/01/2016) | 0.049 | 0.006 | Inf | 8.800 | <0.001 |
| (other 06/16/2016) - (rhod 09/01/2016) | -0.055 | 0.008 | Inf | -6.469 | <0.001 |
| (other 06/16/2016) - (shell 09/01/2016) | -0.015 | 0.008 | Inf | -1.977 | 0.912 |
| (rhod 06/16/2016) - (shell 06/16/2016) | 0.149 | 0.012 | Inf | 12.919 | <0.001 |
| (rhod 06/16/2016) - (kelp 07/10/2016) | 0.076 | 0.012 | Inf | 6.095 | <0.001 |
| (rhod 06/16/2016) - (other 07/10/2016) | 0.179 | 0.011 | Inf | 16.458 | <0.001 |
| (rhod 06/16/2016) - (rhod 07/10/2016) | -0.102 | 0.014 | Inf | -7.428 | <0.001 |
| (rhod 06/16/2016) - (shell 07/10/2016) | 0.138 | 0.012 | Inf | 11.898 | <0.001 |
| (rhod 06/16/2016) - (kelp 08/08/2016) | 0.175 | 0.011 | Inf | 16.606 | <0.001 |
| (rhod 06/16/2016) - (other 08/08/2016) | 0.070 | 0.012 | Inf | 5.699 | <0.001 |
| (rhod 06/16/2016) - (rhod 08/08/2016) | -0.096 | 0.013 | Inf | -7.263 | <0.001 |
| (rhod 06/16/2016) - (shell 08/08/2016) | 0.098 | 0.012 | Inf | 8.203 | <0.001 |
| (rhod 06/16/2016) - (kelp 09/01/2016) | 0.169 | 0.011 | Inf | 15.811 | <0.001 |
| (rhod 06/16/2016) - (other 09/01/2016) | 0.214 | 0.010 | Inf | 21.129 | <0.001 |
| (rhod 06/16/2016) - (rhod 09/01/2016) | 0.110 | 0.012 | Inf | 9.539 | <0.001 |
| (rhod 06/16/2016) - (shell 09/01/2016) | 0.150 | 0.011 | Inf | 13.258 | <0.001 |
| (shell 06/16/2016) - (kelp 07/10/2016) | -0.073 | 0.010 | Inf | -7.664 | <0.001 |
| (shell 06/16/2016) - (other 07/10/2016) | 0.030 | 0.007 | Inf | 4.009 | 0.009 |
| (shell 06/16/2016) - (rhod 07/10/2016) | -0.251 | 0.012 | Inf | -21.131 | <0.001 |
| (shell 06/16/2016) - (shell 07/10/2016) | -0.011 | 0.008 | Inf | -1.392 | 0.998 |
| (shell 06/16/2016) - (kelp 08/08/2016) | 0.025 | 0.007 | Inf | 3.698 | 0.030 |
| (shell 06/16/2016) - (other 08/08/2016) | -0.079 | 0.009 | Inf | -8.346 | <0.001 |
| (shell 06/16/2016) - (rhod 08/08/2016) | -0.245 | 0.011 | Inf | -21.684 | <0.001 |
| (shell 06/16/2016) - (shell 08/08/2016) | -0.051 | 0.008 | Inf | -6.201 | <0.001 |
| (shell 06/16/2016) - (kelp 09/01/2016) | 0.020 | 0.007 | Inf | 2.797 | 0.358 |
| (shell 06/16/2016) - (other 09/01/2016) | 0.065 | 0.006 | Inf | 10.356 | <0.001 |
| (shell 06/16/2016) - (rhod 09/01/2016) | -0.039 | 0.009 | Inf | -4.415 | 0.002 |
| (shell 06/16/2016) - (shell 09/01/2016) | 0.001 | 0.008 | Inf | 0.086 | 1.000 |
| (kelp 07/10/2016) - (other 07/10/2016) | 0.103 | 0.009 | Inf | 11.793 | <0.001 |
| (kelp 07/10/2016) - (rhod 07/10/2016) | -0.178 | 0.013 | Inf | -13.945 | <0.001 |
| (kelp 07/10/2016) - (shell 07/10/2016) | 0.062 | 0.010 | Inf | 6.455 | <0.001 |
| (kelp 07/10/2016) - (kelp 08/08/2016) | 0.099 | 0.007 | Inf | 13.692 | <0.001 |
| (kelp 07/10/2016) - (other 08/08/2016) | -0.005 | 0.011 | Inf | -0.520 | 1.000 |
| (kelp 07/10/2016) - (rhod 08/08/2016) | -0.172 | 0.012 | Inf | -14.067 | <0.001 |
| (kelp 07/10/2016) - (shell 08/08/2016) | 0.022 | 0.010 | Inf | 2.185 | 0.810 |
| (kelp 07/10/2016) - (kelp 09/01/2016) | 0.093 | 0.007 | Inf | 12.512 | <0.001 |
| (kelp 07/10/2016) - (other 09/01/2016) | 0.138 | 0.008 | Inf | 17.723 | <0.001 |
| (kelp 07/10/2016) - (rhod 09/01/2016) | 0.034 | 0.010 | Inf | 3.413 | 0.076 |
| (kelp 07/10/2016) - (shell 09/01/2016) | 0.074 | 0.009 | Inf | 7.982 | <0.001 |
| (other 07/10/2016) - (rhod 07/10/2016) | -0.281 | 0.011 | Inf | -25.010 | <0.001 |
| (other 07/10/2016) - (shell 07/10/2016) | -0.041 | 0.007 | Inf | -5.452 | <0.001 |
| (other 07/10/2016) - (kelp 08/08/2016) | -0.004 | 0.006 | Inf | -0.755 | 1.000 |
| (other 07/10/2016) - (other 08/08/2016) | -0.108 | 0.008 | Inf | -13.846 | <0.001 |
| (other 07/10/2016) - (rhod 08/08/2016) | -0.275 | 0.011 | Inf | -25.897 | <0.001 |
| (other 07/10/2016) - (shell 08/08/2016) | -0.081 | 0.008 | Inf | -10.199 | <0.001 |
| (other 07/10/2016) - (kelp 09/01/2016) | -0.010 | 0.006 | Inf | -1.613 | 0.988 |
| (other 07/10/2016) - (other 09/01/2016) | 0.035 | 0.005 | Inf | 7.374 | <0.001 |
| (other 07/10/2016) - (rhod 09/01/2016) | -0.069 | 0.008 | Inf | -8.643 | <0.001 |
| (other 07/10/2016) - (shell 09/01/2016) | -0.029 | 0.007 | Inf | -4.139 | 0.006 |
| (rhod 07/10/2016) - (shell 07/10/2016) | 0.240 | 0.012 | Inf | 20.104 | <0.001 |
| (rhod 07/10/2016) - (kelp 08/08/2016) | 0.277 | 0.011 | Inf | 25.403 | <0.001 |
| (rhod 07/10/2016) - (other 08/08/2016) | 0.173 | 0.013 | Inf | 13.616 | <0.001 |
| (rhod 07/10/2016) - (rhod 08/08/2016) | 0.006 | 0.013 | Inf | 0.459 | 1.000 |
| (rhod 07/10/2016) - (shell 08/08/2016) | 0.200 | 0.012 | Inf | 16.320 | <0.001 |
| (rhod 07/10/2016) - (kelp 09/01/2016) | 0.271 | 0.011 | Inf | 24.501 | <0.001 |
| (rhod 07/10/2016) - (other 09/01/2016) | 0.316 | 0.011 | Inf | 30.039 | <0.001 |
| (rhod 07/10/2016) - (rhod 09/01/2016) | 0.212 | 0.012 | Inf | 18.140 | <0.001 |
| (rhod 07/10/2016) - (shell 09/01/2016) | 0.252 | 0.012 | Inf | 21.619 | <0.001 |
| (shell 07/10/2016) - (kelp 08/08/2016) | 0.037 | 0.007 | Inf | 5.250 | <0.001 |
| (shell 07/10/2016) - (other 08/08/2016) | -0.068 | 0.010 | Inf | -7.115 | <0.001 |
| (shell 07/10/2016) - (rhod 08/08/2016) | -0.234 | 0.011 | Inf | -20.597 | <0.001 |
| (shell 07/10/2016) - (shell 08/08/2016) | -0.040 | 0.008 | Inf | -5.010 | <0.001 |
| (shell 07/10/2016) - (kelp 09/01/2016) | 0.031 | 0.007 | Inf | 4.302 | 0.003 |
| (shell 07/10/2016) - (other 09/01/2016) | 0.076 | 0.006 | Inf | 11.941 | <0.001 |
| (shell 07/10/2016) - (rhod 09/01/2016) | -0.028 | 0.009 | Inf | -3.134 | 0.166 |
| (shell 07/10/2016) - (shell 09/01/2016) | 0.012 | 0.008 | Inf | 1.568 | 0.992 |
| (kelp 08/08/2016) - (other 08/08/2016) | -0.104 | 0.008 | Inf | -12.794 | <0.001 |
| (kelp 08/08/2016) - (rhod 08/08/2016) | -0.271 | 0.010 | Inf | -26.395 | <0.001 |
| (kelp 08/08/2016) - (shell 08/08/2016) | -0.077 | 0.007 | Inf | -10.299 | <0.001 |
| (kelp 08/08/2016) - (kelp 09/01/2016) | -0.005 | 0.005 | Inf | -1.134 | 1.000 |
| (kelp 08/08/2016) - (other 09/01/2016) | 0.039 | 0.004 | Inf | 9.749 | <0.001 |
| (kelp 08/08/2016) - (rhod 09/01/2016) | -0.065 | 0.007 | Inf | -8.639 | <0.001 |
| (kelp 08/08/2016) - (shell 09/01/2016) | -0.025 | 0.006 | Inf | -3.839 | 0.018 |
| (other 08/08/2016) - (rhod 08/08/2016) | -0.167 | 0.012 | Inf | -13.729 | <0.001 |
| (other 08/08/2016) - (shell 08/08/2016) | 0.027 | 0.010 | Inf | 2.765 | 0.380 |
| (other 08/08/2016) - (kelp 09/01/2016) | 0.099 | 0.008 | Inf | 11.784 | <0.001 |
| (other 08/08/2016) - (other 09/01/2016) | 0.144 | 0.007 | Inf | 19.314 | <0.001 |
| (other 08/08/2016) - (rhod 09/01/2016) | 0.040 | 0.010 | Inf | 4.007 | 0.009 |
| (other 08/08/2016) - (shell 09/01/2016) | 0.079 | 0.009 | Inf | 8.693 | <0.001 |
| (rhod 08/08/2016) - (shell 08/08/2016) | 0.194 | 0.012 | Inf | 16.591 | <0.001 |
| (rhod 08/08/2016) - (kelp 09/01/2016) | 0.265 | 0.011 | Inf | 25.395 | <0.001 |
| (rhod 08/08/2016) - (other 09/01/2016) | 0.310 | 0.010 | Inf | 31.444 | <0.001 |
| (rhod 08/08/2016) - (rhod 09/01/2016) | 0.206 | 0.011 | Inf | 18.730 | <0.001 |
| (rhod 08/08/2016) - (shell 09/01/2016) | 0.246 | 0.011 | Inf | 22.234 | <0.001 |
| (shell 08/08/2016) - (kelp 09/01/2016) | 0.071 | 0.008 | Inf | 9.251 | <0.001 |
| (shell 08/08/2016) - (other 09/01/2016) | 0.116 | 0.007 | Inf | 16.826 | <0.001 |
| (shell 08/08/2016) - (rhod 09/01/2016) | 0.012 | 0.009 | Inf | 1.318 | 0.999 |
| (shell 08/08/2016) - (shell 09/01/2016) | 0.052 | 0.008 | Inf | 6.733 | <0.001 |
| (kelp 09/01/2016) - (other 09/01/2016) | 0.045 | 0.005 | Inf | 9.943 | <0.001 |
| (kelp 09/01/2016) - (rhod 09/01/2016) | -0.059 | 0.008 | Inf | -7.650 | <0.001 |
| (kelp 09/01/2016) - (shell 09/01/2016) | -0.019 | 0.007 | Inf | -2.867 | 0.310 |
| (other 09/01/2016) - (rhod 09/01/2016) | -0.104 | 0.007 | Inf | -15.025 | <0.001 |
| (other 09/01/2016) - (shell 09/01/2016) | -0.064 | 0.006 | Inf | -11.073 | <0.001 |
| (rhod 09/01/2016) - (shell 09/01/2016) | 0.040 | 0.009 | Inf | 4.659 | 0.001 |

**Table B5.** Tukey tests on the differences in frequency of covering in green sea urchin (*Strongylocentrotus droebachiensis*) with the four different types of covering materials considered across sampling depths.

| Comparison | Difference | Std. Error | *df* | z-ratio | *p*(>*z*) |
| --- | --- | --- | --- | --- | --- |
|  |  |  |  |  |  |
| kelp Deep - other Deep | -0.020 | 0.003 | Inf | -6.041 | <0.001 |
| kelp Deep - rhod Deep | -0.316 | 0.009 | Inf | -36.566 | <0.001 |
| kelp Deep - shell Deep | -0.020 | 0.003 | Inf | -6.263 | <0.001 |
| kelp Deep - kelp Shallow | -0.152 | 0.007 | Inf | -22.247 | <0.001 |
| kelp Deep - other Shallow | -0.154 | 0.006 | Inf | -24.148 | <0.001 |
| kelp Deep - rhod Shallow | -0.131 | 0.006 | Inf | -22.387 | <0.001 |
| kelp Deep - shell Shallow | -0.184 | 0.007 | Inf | -25.719 | <0.001 |
| kelp Deep - kelp Int | -0.003 | 0.002 | Inf | -1.682 | 0.877 |
| kelp Deep - other Int | -0.039 | 0.004 | Inf | -11.224 | <0.001 |
| kelp Deep - rhod Int | -0.228 | 0.007 | Inf | -32.310 | <0.001 |
| kelp Deep - shell Int | -0.076 | 0.005 | Inf | -15.872 | <0.001 |
| other Deep - rhod Deep | -0.296 | 0.009 | Inf | -32.694 | <0.001 |
| other Deep - shell Deep | <0.001 | 0.004 | Inf | 0.041 | 1.000 |
| other Deep - kelp Shallow | -0.132 | 0.007 | Inf | -17.855 | <0.001 |
| other Deep - other Shallow | -0.134 | 0.007 | Inf | -19.814 | <0.001 |
| other Deep - rhod Shallow | -0.111 | 0.006 | Inf | -17.230 | <0.001 |
| other Deep - shell Shallow | -0.164 | 0.008 | Inf | -21.459 | <0.001 |
| other Deep - kelp Int | 0.017 | 0.003 | Inf | 5.101 | <0.001 |
| other Deep - other Int | -0.019 | 0.004 | Inf | -4.484 | 0.001 |
| other Deep - rhod Int | -0.208 | 0.008 | Inf | -27.530 | <0.001 |
| other Deep - shell Int | -0.056 | 0.005 | Inf | -10.157 | <0.001 |
| rhod Deep - shell Deep | 0.296 | 0.009 | Inf | 32.901 | <0.001 |
| rhod Deep - kelp Shallow | 0.164 | 0.011 | Inf | 15.091 | <0.001 |
| rhod Deep - other Shallow | 0.162 | 0.011 | Inf | 15.336 | <0.001 |
| rhod Deep - rhod Shallow | 0.185 | 0.010 | Inf | 18.637 | <0.001 |
| rhod Deep - shell Shallow | 0.132 | 0.011 | Inf | 11.901 | <0.001 |
| rhod Deep - kelp Int | 0.313 | 0.009 | Inf | 36.214 | <0.001 |
| rhod Deep - other Int | 0.277 | 0.009 | Inf | 30.292 | <0.001 |
| rhod Deep - rhod Int | 0.088 | 0.011 | Inf | 8.396 | <0.001 |
| rhod Deep - shell Int | 0.240 | 0.010 | Inf | 24.833 | <0.001 |
| shell Deep - kelp Shallow | -0.132 | 0.007 | Inf | -18.034 | <0.001 |
| shell Deep - other Shallow | -0.134 | 0.007 | Inf | -19.565 | <0.001 |
| shell Deep - rhod Shallow | -0.111 | 0.006 | Inf | -17.454 | <0.001 |
| shell Deep - shell Shallow | -0.164 | 0.007 | Inf | -22.103 | <0.001 |
| shell Deep - kelp Int | 0.017 | 0.003 | Inf | 5.281 | <0.001 |
| shell Deep - other Int | -0.020 | 0.004 | Inf | -4.533 | <0.001 |
| shell Deep - rhod Int | -0.208 | 0.007 | Inf | -27.781 | <0.001 |
| shell Deep - shell Int | -0.056 | 0.005 | Inf | -10.593 | <0.001 |
| kelp Shallow - other Shallow | -0.002 | 0.009 | Inf | -0.226 | 1.000 |
| kelp Shallow - rhod Shallow | 0.021 | 0.009 | Inf | 2.353 | 0.439 |
| kelp Shallow - shell Shallow | -0.032 | 0.010 | Inf | -3.332 | 0.041 |
| kelp Shallow - kelp Int | 0.149 | 0.007 | Inf | 22.173 | <0.001 |
| kelp Shallow - other Int | 0.112 | 0.007 | Inf | 15.034 | <0.001 |
| kelp Shallow - rhod Int | -0.076 | 0.010 | Inf | -7.876 | <0.001 |
| kelp Shallow - shell Int | 0.076 | 0.008 | Inf | 9.367 | <0.001 |
| other Shallow - rhod Shallow | 0.023 | 0.008 | Inf | 2.706 | 0.223 |
| other Shallow - shell Shallow | -0.030 | 0.009 | Inf | -3.234 | 0.056 |
| other Shallow - kelp Int | 0.151 | 0.006 | Inf | 23.670 | <0.001 |
| other Shallow - other Int | 0.114 | 0.006 | Inf | 17.902 | <0.001 |
| other Shallow - rhod Int | -0.074 | 0.009 | Inf | -7.948 | <0.001 |
| other Shallow - shell Int | 0.078 | 0.008 | Inf | 10.142 | <0.001 |
| rhod Shallow - shell Shallow | -0.053 | 0.009 | Inf | -5.879 | <0.001 |
| rhod Shallow - kelp Int | 0.128 | 0.006 | Inf | 21.866 | <0.001 |
| rhod Shallow - other Int | 0.092 | 0.007 | Inf | 13.985 | <0.001 |
| rhod Shallow - rhod Int | -0.097 | 0.008 | Inf | -11.949 | <0.001 |
| rhod Shallow - shell Int | 0.055 | 0.007 | Inf | 7.600 | <0.001 |
| shell Shallow - kelp Int | 0.181 | 0.007 | Inf | 25.293 | <0.001 |
| shell Shallow - other Int | 0.145 | 0.008 | Inf | 18.701 | <0.001 |
| shell Shallow - rhod Int | -0.044 | 0.010 | Inf | -4.420 | 0.001 |
| shell Shallow - shell Int | 0.109 | 0.007 | Inf | 14.547 | <0.001 |
| kelp Int - other Int | -0.036 | 0.004 | Inf | -10.349 | <0.001 |
| kelp Int - rhod Int | -0.225 | 0.007 | Inf | -31.880 | <0.001 |
| kelp Int - shell Int | -0.072 | 0.005 | Inf | -15.229 | <0.001 |
| other Int - rhod Int | -0.189 | 0.008 | Inf | -24.659 | <0.001 |
| other Int - shell Int | -0.036 | 0.006 | Inf | -6.461 | <0.001 |
| rhod Int - shell Int | 0.152 | 0.008 | Inf | 18.368 | <0.001 |

**Table B6.** Tukey tests on the differences in frequency of covering in green sea urchin (*Strongylocentrotus droebachiensis*) with the four different types of covering materials considered across sea urchin body size classes [A = [1-2[, B = [2-3[, C = [3-4[, D = [4-5[ cm t.d.).

| Comparison | Difference | Std. Error | *df* | z-ratio | *p*(>*z*) |
| --- | --- | --- | --- | --- | --- |
| kelp D - other D | 0.015 | 0.006 | Inf | 2.591 | 0.405 |
| kelp D - rhod D | -0.079 | 0.009 | Inf | -9.181 | <0.001 |
| kelp D - shell D | 0.010 | 0.006 | Inf | 1.762 | 0.930 |
| kelp D - kelp A | 0.060 | 0.009 | Inf | 6.962 | <0.001 |
| kelp D - other A | -0.007 | 0.010 | Inf | -0.716 | 1.000 |
| kelp D - rhod A | -0.238 | 0.015 | Inf | -16.183 | <0.001 |
| kelp D - shell A | -0.041 | 0.011 | Inf | -3.556 | 0.033 |
| kelp D - kelp B | 0.039 | 0.005 | Inf | 7.269 | <0.001 |
| kelp D - other B | 0.018 | 0.006 | Inf | 3.073 | 0.140 |
| kelp D - rhod B | -0.143 | 0.008 | Inf | -17.862 | <0.001 |
| kelp D - shell B | -0.014 | 0.007 | Inf | -2.203 | 0.695 |
| kelp D - kelp C | 0.019 | 0.005 | Inf | 4.215 | 0.003 |
| kelp D - other C | 0.015 | 0.005 | Inf | 3.028 | 0.157 |
| kelp D - rhod C | -0.115 | 0.007 | Inf | -16.967 | <0.001 |
| kelp D - shell C | -0.004 | 0.005 | Inf | -0.717 | 1.000 |
| other D - rhod D | -0.094 | 0.009 | Inf | -10.650 | <0.001 |
| other D - shell D | -0.004 | 0.006 | Inf | -0.685 | 1.000 |
| other D - kelp A | 0.045 | 0.009 | Inf | 5.183 | <0.001 |
| other D - other A | -0.022 | 0.010 | Inf | -2.116 | 0.755 |
| other D - rhod A | -0.253 | 0.015 | Inf | -17.048 | <0.001 |
| other D - shell A | -0.055 | 0.012 | Inf | -4.776 | <0.001 |
| other D - kelp B | 0.025 | 0.006 | Inf | 4.356 | 0.001 |
| other D - other B | 0.003 | 0.006 | Inf | 0.540 | 1.000 |
| other D - rhod B | -0.158 | 0.008 | Inf | -19.208 | <0.001 |
| other D - shell B | -0.029 | 0.007 | Inf | -4.283 | 0.002 |
| other D - kelp C | 0.004 | 0.005 | Inf | 0.900 | 1.000 |
| other D - other C | <0.001 | 0.005 | Inf | 0.027 | 1.000 |
| other D - rhod C | -0.129 | 0.007 | Inf | -18.488 | <0.001 |
| other D - shell C | -0.018 | 0.005 | Inf | -3.360 | 0.062 |
| rhod D - shell D | 0.089 | 0.009 | Inf | 9.980 | <0.001 |
| rhod D - kelp A | 0.139 | 0.011 | Inf | 12.780 | <0.001 |
| rhod D - other A | 0.072 | 0.012 | Inf | 6.045 | <0.001 |
| rhod D - rhod A | -0.159 | 0.017 | Inf | -9.597 | <0.001 |
| rhod D - shell A | 0.038 | 0.013 | Inf | 2.891 | 0.219 |
| rhod D - kelp B | 0.118 | 0.009 | Inf | 13.720 | <0.001 |
| rhod D - other B | 0.097 | 0.009 | Inf | 10.869 | <0.001 |
| rhod D - rhod B | -0.064 | 0.011 | Inf | -6.105 | <0.001 |
| rhod D - shell B | 0.065 | 0.009 | Inf | 6.887 | <0.001 |
| rhod D - kelp C | 0.098 | 0.008 | Inf | 12.032 | <0.001 |
| rhod D - other C | 0.094 | 0.008 | Inf | 11.271 | <0.001 |
| rhod D - rhod C | -0.036 | 0.009 | Inf | -3.894 | 0.010 |
| rhod D - shell C | 0.075 | 0.008 | Inf | 8.880 | <0.001 |
| shell D - kelp A | 0.049 | 0.009 | Inf | 5.561 | <0.001 |
| shell D - other A | -0.017 | 0.010 | Inf | -1.720 | 0.942 |
| shell D - rhod A | -0.249 | 0.015 | Inf | -16.652 | <0.001 |
| shell D - shell A | -0.051 | 0.012 | Inf | -4.282 | 0.002 |
| shell D - kelp B | 0.029 | 0.006 | Inf | 4.881 | <0.001 |
| shell D - other B | 0.008 | 0.006 | Inf | 1.190 | 0.999 |
| shell D - rhod B | -0.154 | 0.008 | Inf | -18.298 | <0.001 |
| shell D - shell B | -0.025 | 0.007 | Inf | -3.509 | 0.038 |
| shell D - kelp C | 0.009 | 0.005 | Inf | 1.660 | 0.957 |
| shell D - other C | 0.004 | 0.005 | Inf | 0.796 | 1.000 |
| shell D - rhod C | -0.125 | 0.007 | Inf | -17.365 | <0.001 |
| shell D - shell C | -0.014 | 0.006 | Inf | -2.523 | 0.454 |
| kelp A - other A | -0.067 | 0.012 | Inf | -5.634 | <0.001 |
| kelp A - rhod A | -0.298 | 0.016 | Inf | -18.460 | <0.001 |
| kelp A - shell A | -0.100 | 0.013 | Inf | -7.596 | <0.001 |
| kelp A - kelp B | -0.020 | 0.008 | Inf | -2.397 | 0.550 |
| kelp A - other B | -0.042 | 0.009 | Inf | -4.728 | <0.001 |
| kelp A - rhod B | -0.203 | 0.010 | Inf | -19.531 | <0.001 |
| kelp A - shell B | -0.074 | 0.009 | Inf | -7.971 | <0.001 |
| kelp A - kelp C | -0.041 | 0.008 | Inf | -5.036 | <0.001 |
| kelp A - other C | -0.045 | 0.008 | Inf | -5.470 | <0.001 |
| kelp A - rhod C | -0.174 | 0.009 | Inf | -18.444 | <0.001 |
| kelp A - shell C | -0.063 | 0.008 | Inf | -7.564 | <0.001 |
| other A - rhod A | -0.231 | 0.017 | Inf | -13.714 | <0.001 |
| other A - shell A | -0.034 | 0.014 | Inf | -2.387 | 0.558 |
| other A - kelp B | 0.046 | 0.010 | Inf | 4.712 | <0.001 |
| other A - other B | 0.025 | 0.010 | Inf | 2.495 | 0.475 |
| other A - rhod B | -0.136 | 0.012 | Inf | -11.865 | <0.001 |
| other A - shell B | -0.007 | 0.011 | Inf | -0.700 | 1.000 |
| other A - kelp C | 0.026 | 0.009 | Inf | 2.767 | 0.288 |
| other A - other C | 0.022 | 0.010 | Inf | 2.256 | 0.657 |
| other A - rhod C | -0.108 | 0.011 | Inf | -10.108 | <0.001 |
| other A - shell C | 0.003 | 0.010 | Inf | 0.343 | 1.000 |
| rhod A - shell A | 0.198 | 0.018 | Inf | 11.077 | <0.001 |
| rhod A - kelp B | 0.278 | 0.015 | Inf | 18.836 | <0.001 |
| rhod A - other B | 0.256 | 0.015 | Inf | 17.183 | <0.001 |
| rhod A - rhod B | 0.095 | 0.016 | Inf | 6.006 | <0.001 |
| rhod A - shell B | 0.224 | 0.015 | Inf | 14.740 | <0.001 |
| rhod A - kelp C | 0.257 | 0.015 | Inf | 17.791 | <0.001 |
| rhod A - other C | 0.253 | 0.015 | Inf | 17.379 | <0.001 |
| rhod A - rhod C | 0.124 | 0.016 | Inf | 7.963 | <0.001 |
| rhod A - shell C | 0.235 | 0.015 | Inf | 16.011 | <0.001 |
| shell A - kelp B | 0.080 | 0.011 | Inf | 6.986 | <0.001 |
| shell A - other B | 0.059 | 0.012 | Inf | 5.021 | <0.001 |
| shell A - rhod B | -0.103 | 0.013 | Inf | -7.969 | <0.001 |
| shell A - shell B | 0.026 | 0.012 | Inf | 2.202 | 0.696 |
| shell A - kelp C | 0.060 | 0.011 | Inf | 5.382 | <0.001 |
| shell A - other C | 0.055 | 0.011 | Inf | 4.939 | <0.001 |
| shell A - rhod C | -0.074 | 0.012 | Inf | -6.092 | <0.001 |
| shell A - shell C | 0.037 | 0.011 | Inf | 3.232 | 0.090 |
| kelp B - other B | -0.021 | 0.006 | Inf | -3.640 | 0.025 |
| kelp B - rhod B | -0.183 | 0.008 | Inf | -22.704 | <0.001 |
| kelp B - shell B | -0.054 | 0.007 | Inf | -8.196 | <0.001 |
| kelp B - kelp C | -0.020 | 0.005 | Inf | -4.384 | 0.001 |
| kelp B - other C | -0.025 | 0.005 | Inf | -5.009 | <0.001 |
| kelp B - rhod C | -0.154 | 0.007 | Inf | -22.710 | <0.001 |
| kelp B - shell C | -0.043 | 0.005 | Inf | -8.311 | <0.001 |
| other B - rhod B | -0.161 | 0.008 | Inf | -19.288 | <0.001 |
| other B - shell B | -0.032 | 0.007 | Inf | -4.660 | <0.001 |
| other B - kelp C | 0.001 | 0.005 | Inf | 0.212 | 1.000 |
| other B - other C | -0.003 | 0.005 | Inf | -0.589 | 1.000 |
| other B - rhod C | -0.132 | 0.007 | Inf | -18.535 | <0.001 |
| other B - shell C | -0.022 | 0.006 | Inf | -3.826 | 0.013 |
| rhod B - shell B | 0.129 | 0.009 | Inf | 14.569 | <0.001 |
| rhod B - kelp C | 0.162 | 0.008 | Inf | 21.552 | <0.001 |
| rhod B - other C | 0.158 | 0.008 | Inf | 20.485 | <0.001 |
| rhod B - rhod C | 0.029 | 0.009 | Inf | 3.178 | 0.105 |
| rhod B - shell C | 0.140 | 0.008 | Inf | 17.697 | <0.001 |
| shell B - kelp C | 0.033 | 0.006 | Inf | 5.654 | <0.001 |
| shell B - other C | 0.029 | 0.006 | Inf | 4.742 | <0.001 |
| shell B - rhod C | -0.100 | 0.008 | Inf | -12.983 | <0.001 |
| shell B - shell C | 0.011 | 0.006 | Inf | 1.670 | 0.955 |
| kelp C - other C | -0.004 | 0.004 | Inf | -1.070 | 1.000 |
| kelp C - rhod C | -0.134 | 0.006 | Inf | -21.685 | <0.001 |
| kelp C - shell C | -0.023 | 0.004 | Inf | -5.242 | <0.001 |
| other C - rhod C | -0.129 | 0.006 | Inf | -20.258 | <0.001 |
| other C - shell C | -0.018 | 0.005 | Inf | -3.967 | 0.007 |
| rhod C - shell C | 0.111 | 0.007 | Inf | 16.817 | <0.001 |
